# Implementing *In Vitro* Bioactivity Data to Modernize Priority Setting of Chemical Inventories

**DOI:** 10.14573/altex.2106171

**Published:** 2021-11-23

**Authors:** Marc A. Beal, Matthew Gagne, Sunil A. Kulkarni, Grace Patlewicz, Russell S. Thomas

**Affiliations:** 1 Healthy Environments and Consumer Safety Branch, Health Canada, Ottawa, Canada; 2 Center for Computational Toxicology and Exposure, Office of Research and Development, U.S. Environmental Protection Agency, Research Triangle Park, NC, USA

## Abstract

Internationally, there are thousands of existing and newly introduced chemicals in commerce, highlighting the ongoing importance of innovative approaches to identify emerging chemicals of concern. For many chemicals, there is a paucity of hazard and exposure data. Thus, there is a crucial need for efficient and robust approaches to address data gaps and support risk-based prioritization. Several studies have demonstrated the utility of *in vitro* bioactivity data from the ToxCast program in deriving points of departure (PODs). ToxCast contains data for nearly 1,400 endpoints per chemical, and the bioactivity concentrations, indicative of potential adverse outcomes, can be converted to human-equivalent PODs using high-throughput toxicokinetics (HTTK) modeling. However, data gaps need to be addressed for broader application: the limited chemical space of HTTK and quantitative high-throughput screening data. Here we explore the applicability of *in silico* models to address these data needs. Specifically, we used ADMET predictor for HTTK predictions and a generalized read-across approach to predict ToxCast bioactivity potency. We applied these models to profile 5,801 chemicals on Canada’s Domestic Substances List (DSL). To evaluate the approach’s performance, bioactivity PODs were compared with *in vivo* results from the EPA Toxicity Values database for 1,042 DSL chemicals. Comparisons demonstrated that the bioactivity PODs, based on ToxCast data or read-across, were conservative for 95% of the chemicals. Comparing bioactivity PODs to human exposure estimates supports the identification of chemicals of potential interest for further work. The bioactivity workflow shows promise as a powerful screening tool to support effective triaging of chemical inventories.

## 1 Introduction

Methods for identifying priorities for chemical risk assessment and risk management serve a critical role in chemicals management systems globally (OECD, 2019). In most jurisdictions, prioritization schemes select from existing inventories of chemicals known to be in commerce for that region. Each chemical inventory is unique to the country or regulatory agency, but there is acknowledgement of the presence of overlapping interests and priorities internationally. For example, under the existing substances risk assessment program of Canada’s Chemicals Management Plan, Health Canada (HC) and Environment and Climate Change Canada (ECCC) focus on evaluating chemicals from the Domestic Substances List (DSL), which contains more than 26,000 chemicals^1^. The approach for the Identification of Risk Assessment Priorities^2^ is a cyclical review process that is conducted by both government departments to identify new scientific evidence on DSL chemicals and higher priority substances for further action. These actions could include risk assessment, risk management, data collection, research and monitoring, or the generation of new data. A common challenge for prioritization efforts, and risk assessment in general, is the lack of exposure or toxicity data available to inform risk. Consequently, chemicals have been traditionally prioritized for assessment based on data sufficiency rather than inherent toxicity and potential risk. Thus, there is a need to leverage emerging technologies for the development of more innovative and modern approaches, capable of addressing both hazard and exposure data gaps, to make prioritization schemes more pragmatic, efficient, transparent, and proactive.

Across the different international agencies, there is increasing demand for data required to support chemical safety and risk assessments, and there is recognition that traditional animal studies alone will be unable to address these data needs (Kavlock et al., 2018). Specifically, animal studies are too prohibitive to efficient resource allocation, and there is a degree of uncertainty associated with the human relevance of results. Moreover, there are global pressures to develop robust and reliable alternatives to animal testing (Kavlock et al., 2018). For these reasons, there are coordinated efforts between international collaborators to modernize approaches for screening, priority setting, and risk assessment by exploring and implementing new approach methodologies (NAMs). NAMs broadly refer to any novel technologies, methods, and/or approaches designed to support risk evaluation that serve to reduce, refine, or replace vertebrate animals. NAM data are diverse and encompass areas such as *in vitro* toxicodynamics and toxicokinetics, exposure science, omics technologies, and computational chemistry. Importantly, *in silico* and experimental NAM data are readily available for thousands of chemicals, most often in public databases. When there are no traditional *in vivo* data available for chemical risk assessment, NAMs can be used to generate data in a high-throughput and high-content manner. As part of collaborative efforts, such as the Accelerating the Pace of Chemical Risk Assessment (APCRA) (Kavlock et al., 2018) initiative and OECD Integrated Approaches to Testing and Assessment (IATA) (Patlewicz et al., 2014), proof-of-concept and transparent case studies are being conducted to build confidence in the science and application of NAM data in various regulatory contexts.

A large-scale retrospective analysis, conducted under APCRA, demonstrated the utility of *in vitro* biological activity (bioactivity) in establishing NAM-based points of departure (PODs) that are conservative relative to *in vivo* PODs based on apical end-points used currently in traditional risk assessments (Paul Friedman et al., 2019). The APCRA study conducted by Paul Friedman et al. succeeded several smaller case studies investigating chemical bioactivity across a broad series of toxicological *in vitro* assays, all probing early biological events implicated in adverse outcome pathways (AOP; Blackwell et al., 2017; Corsi et al., 2019; Gannon et al., 2019; Judson et al., 2011; Paul Friedman et al., 2016; Tilley et al., 2017; Turley et al., 2019; Wetmore et al., 2011, 2013). The APCRA case study leveraged existing data from the intersection of several sources of NAM information mostly available on the EPA CompTox Chemicals Dashboard (Williams et al., 2017). Specifically, bioactivity data were taken from the EPA’s ToxCast/Tox21 collaborative database^3^, which contains quantitative high-throughput screening (qHTS) data for nearly 1,400 toxicological endpoints assessed across approximately 10,000 chemicals (Richard et al., 2016). The high-throughput toxicokinetics (HTTK) tool available as an open source R package was probed for chemicals that had sufficient data to perform *in vitro* to *in vivo* extrapolation (IVIVE) and model administered equivalent doses (AEDs) in mg/kg bw/day (Pearce et al., 2017). This was key to enable comparisons to be made between *in vitro* derived PODs (commonly referred to as POD_NAM_ or POD_Bioactivity_) and traditional PODs (POD_Traditional_), as well as to compare the bioactivity PODs to exposure estimates. Traditional PODs were identified using the ToxValDB, which is a highly structured database containing publicly extracted *in vivo* toxicity data from thousands of studies covering thousands of chemicals (Williams et al., 2017). Lastly, exposure estimates were pulled from ExpoCast (Cohen Hubal et al., 2010; Wambaugh et al., 2013) to establish bioactivity exposure ratios (BERs) and provide a risk estimate. BERs are analogous with margins of exposure used to support regulatory decision-making and are reported as the POD_Bioactivity_ divided by the exposure estimate or as the log_10_BER ratio (log_10_POD_Bioactivity_ - log_10_Exposure). In total, 448 chemicals had the necessary data to derive a POD_Bioactivity_ to facilitate comparisons with POD_Traditional_ values and exposure estimates.

The results of the case study by Paul Friedman et al. (2016) determined that the POD_Bioactivity_ was lower than the POD_Traditional_ for 89% of chemicals. On average, the POD_Bioactivity_ was 100-fold lower than the POD_Traditional_. For the chemicals that had a higher POD_Bioactivity_ than POD_Traditional_, the POD_Bioactivity_ was typically within one order of magnitude (i.e., a factor of 10). A closer inspection revealed that the chemicals that had a higher POD_Bioactivity_ than POD_Traditional_ had an enrichment of structural features related to organophosphates and carbamate insecticides. Thus, these types of chemicals were recommended as potential exclusion criteria for future application of the approach. The POD_Bioactivity_ was also compared against the threshold of toxicological concern (TTC) derived using the TTC decision tree, which is a well-established and conservative *in silico* approach for setting human exposure threshold values for chemicals (Health Canada, 2016; Kroes et al., 2004; Patlewicz et al., 2018). This comparison demonstrated that the POD_Bioactivity_ was higher than the TTC for 90% of the chemicals, indicating that the POD_Bioactivity_ could be useful as part of a tiered risk assessment framework subsequent to the TTC. After establishing confidence in the approach, the POD_Bioactivity_ values were compared against exposure estimates to derive BERs for the purpose of screening chemicals to identify those of greater potential for concern. A key achievement of the APCRA retrospective case study was the development of a generic workflow, offering a trade-off between uncertainty for higher throughput, that could be broadly applied to many different chemical classes.

The BER approach and workflow as described already has promise to be a powerful tool for rapid screening of chemical inventories. However, in order to apply the approach on a larger scale, there are key data gaps that need to be addressed and areas of uncertainty for possible refinement. Specifically, to derive POD_Bioactivity_ values for hazard assessment, chemicals need to have available HTTK data and have been screened in a battery of *in vitro* assays that cover a broad representation of biological space (e.g., ToxCast). Once a POD_Bioactivity_ has been established, human exposure estimates are required to derive BERs for risk-based evaluation.

**Fig. 1:**
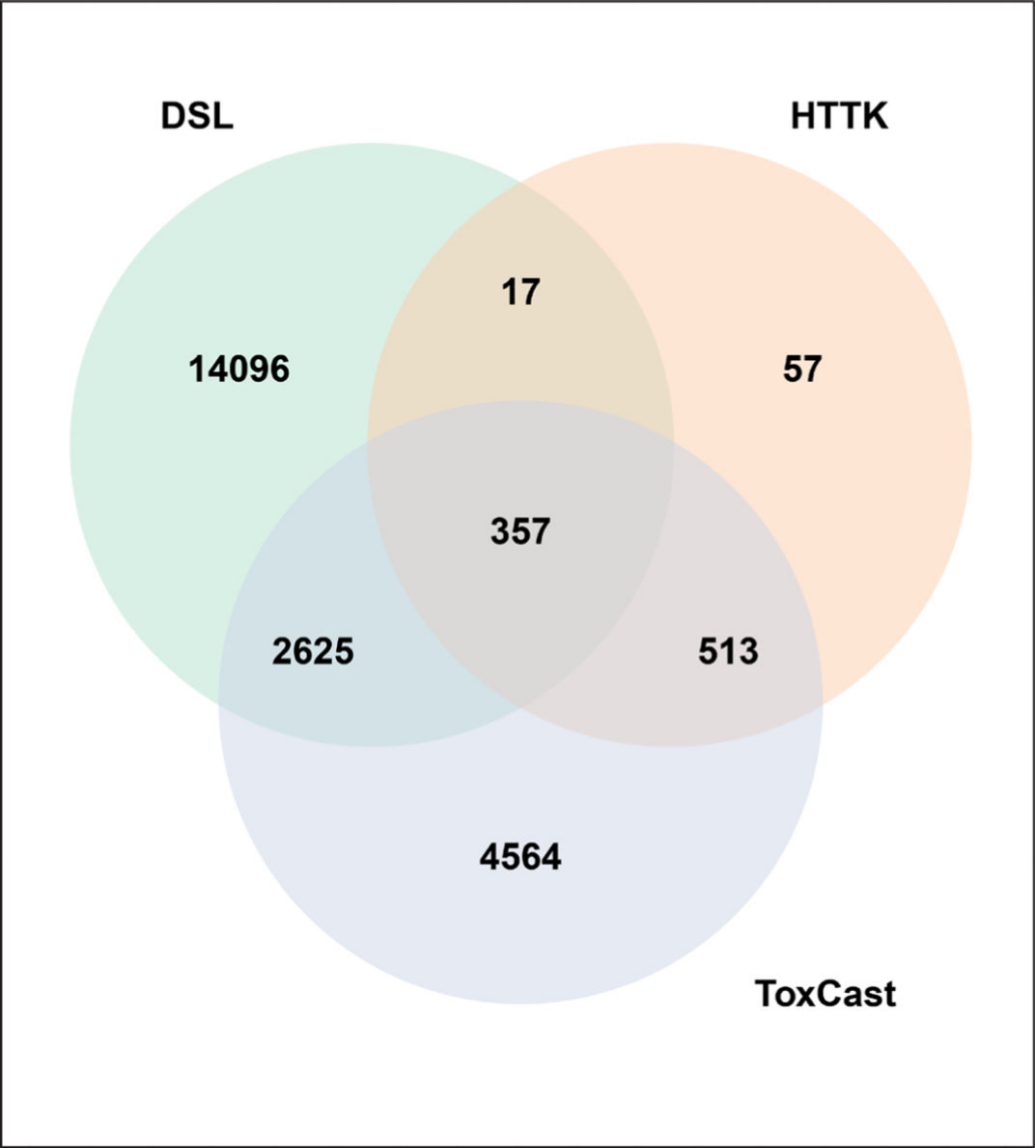
Data gaps for broad BER application The BER approach could be applied for 357 DSL chemicals where there was existing HTTK and ToxCast data. For the remaining chemicals on Canada’s DSL, there are two primary data gaps: 1) an inner gap, where 2,625 chemicals have ToxCast data but no HTTK data, and 2) an outer gap, where 14,096 chemicals have neither HTTK nor ToxCast data.

**Fig. 2:**
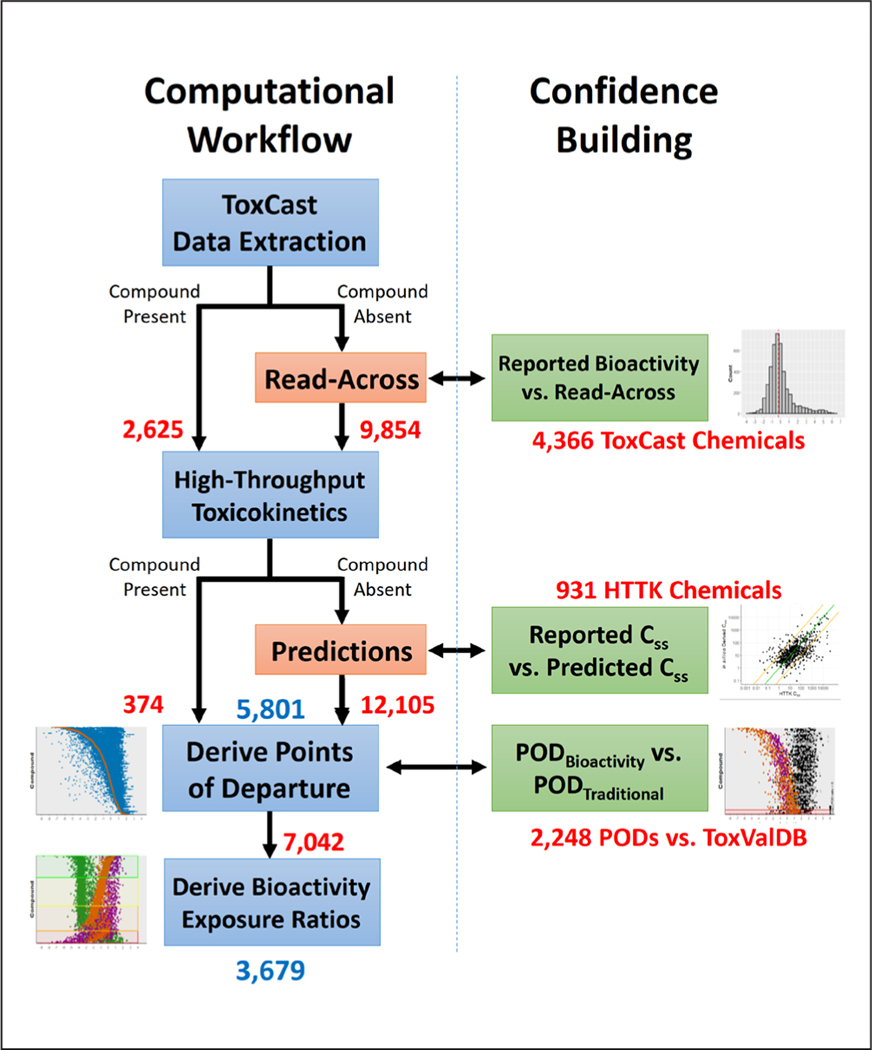
Computational workflow and confidence-building data comparisons The left side of the middle vertical line shows the computational workflow used to derive bioactivity exposure ratios (BERs). Red boxes indicate when a data gap was addressed. Specifically, *in silico* predictions were used to address missing HTTK data and read-across was explored to address chemicals not tested by ToxCast. Green boxes, right of the middle vertical line, indicate where data comparisons were used to assess confidence of data gap-filling and ultimately determine how the NAM-based POD_Bioactivity_ or POD_Read-Across_ values compare to POD_Traditional_ values. The red text indicates the total number of chemicals carried forward at each step of the workflow before the application of any filters. The blue text indicates the total number of chemicals that passed filters and were reported as the final PODs (5,801) or BERs (3,679). The plots displayed next to the workflow steps are explained in more detail in the other figures.

In this proof-of-concept work, we determined the intersection between the Canadian DSL, HTTK, and ToxCast to identify chemicals for which POD_Bioactivity_ values can be derived and to assess the data gaps that need to be addressed for broader application of the approach (Fig. 1). We developed a computational workflow that applied *in silico* predictions and read-across to fill in the HTTK and ToxCast bioactivity data gaps, respectively (Fig. 2). Through this approach, we were able to successfully expand the application of the generic bioactivity workflow from an initial 357 chemicals meeting the minimum data requirements to thousands of chemicals, most of which had one or more data gaps addressed to support application of the approach. This work demonstrates the power of using NAMs combined with read-across methods to triage chemicals of higher potential concern, allowing for more concentrated focus on testing and assessment efforts on chemicals demonstrating the highest potential for hazard and risk. Moreover, effective use of NAMs to narrow the focus of chemical risk assessment activities will support the reduction of animal use in toxicity testing and assessment.

## 2 Materials and methods

### Approach overview and computational workflow

The computational workflow (Fig. 2) closely follows the methods developed by Paul Friedman et al. (2019) and applied in the Science Approach Document developed by Health Canada (2021). Briefly, ToxCast bioactivity data based on AC_50_ values in μM were extracted from the SQL database, inactive assays were filtered out, and from the remaining data, the 5^th^ percentile bioactivity concentration for each chemical was reported. IVIVE was then performed using HTTK to derive AEDs in mg/kg bw/day (i.e., POD_Bioactivity_). For chemicals lacking HTTK data, *in silico* predictions of toxicokinetics parameters were used. For chemicals lacking ToxCast data, generalized read-across (GenRA) using different chemical fingerprint representations was applied to predict bioactivity concentrations for chemicals. The AEDs for read-across chemicals are referred to as the POD_Read-Across_. Comparisons were made in order to corroborate results and build confidence in the *in silico* data gap-filling approaches. Specifically, existing HTTK-derived steady state plasma concentrations (C_ss_ ) were compared with the *in silico*-derived C_ss_ values, and existing ToxCast bioactivity concentrations were compared to bioactivity concentrations derived from the read-across model. Furthermore, the POD_Read-Across_ values, based on both *in silico* HTTK data and read-across, were compared against the true POD_Bioactivity_ where possible (i.e., POD derived using *in vitro* HTTK data and ToxCast bioactivity data).

The methods are presented below in the order that each data gap was addressed. Specifically, the HTTK (inner) data gap was addressed first, and the ToxCast (outer) data gap was addressed second (Fig. 1). This reflects the increasing uncertainty with addressing data gaps, as the majority of chemicals outside the scope of ToxCast also lack HTTK data (i.e., POD_Read-Across_ Uncertainty > POD_Bioactivity_ Uncertainty).

The workflow was mainly performed using the R programming language^4^ (version 2.15), with each exception noted below. All of the code used to analyze and report the data as well as build confidence in the approach is available as a supplementary RMarkdown report, and a tool to derive POD_Bioactivity_ and POD_Read-Across_ is available as an RShiny web-application^5^. The data used in the workflow are either available on public databases or are included in the supplementary material^6^ to allow for reproducibility of results. The results and output of the workflow (i.e., chemical info, PODs, etc.) are provided in the supplementary material^6^.

### Extract bioactivity data from ToxCast database

The methods for this step are described in greater detail else-where (Paul Friedman et al., 2019) and are only briefly discussed here. The *in vitro* bioactivity data for all chemicals in the local install MySQL ToxCast database (invitrodb_v3) (US EPA, 2015) were queried using the ToxCast Data Analysis Pipeline (tcpl) v2.0 R package (Filer et al., 2016). Specifically, levels 5, 6, and 7 data were extracted from the MySQL Tox-Cast database for each chemical tested. Level 5 data contains the hit call information for the assay endpoints of each chemical and the AC_50_ values from the selected concentration-response models used during the curve fitting process. Level 5 data were filtered to only include assay endpoints with an active hit call and endpoints tested in a multiple concentration format. Level 6 and 7 data provide caution flags and uncertainty information, respectively, for curve fits and hit calls. The data were filtered to remove assays with at least three caution flags and a hit percent of less than 50% (only assays meeting both criteria were filtered out). The data were further filtered to remove assays with curve fittings meeting categories 36 and 45, which correspond to AC_50_ values above the maximum tested concentration based on the hill and gain-loss models, respectively. After filtering, the AC_50_ concentrations for each chemical could be used to derive an AED, but only one AED per chemical was reported as the final POD_Bioactivity_. Specifically, the 5^th^ percentile of AC_50_ concentrations for each chemical was carried forward for the derivation of POD_Bioactivity_ values. For chemicals where there were no active AC_50_ concentrations, the maximum concentration tested in ToxCast of 100 μM was carried forward for POD derivation.

### High-throughput toxicokinetics modeling

IVIVE modeling of AC_50_ concentrations in μM to AEDs in mg/kg bw/day was performed using the HTTK package v1.10 (Pearce et al., 2017) in R. Specifically, the three compartment steady-state model (“3compartmentss”), modified from Wetmore et al. (2011, 2015), was used to calculate the C_ss_ at a constant dose rate of 1 mg/kg bw/day. The three compartments consist of the gut, liver, and the rest of the body. At steady state, the plasma concentration is assumed to increase in a linear fashion as the dose rate increases. Using this linear assumption, the AED/AC_50_ ratio is determined to be directly proportional to the constant dose rate divided by the C_ss_. The IVIVE process models the dose rate (AED) that is required to achieve a C_ss_ equal to the AC_50_ concentration. Based on the linear assumption, the following formula can be used to calculate the AED (i.e., POD_Bioactivity_):

(Eq. 1) $$AED=bioactivity\;concentration\;\;(\mu M)\times \frac{\frac{1\frac{mg}{kg}}{day}}{C_{ss}(\mu M)}$$

The HTTK 3compartmentss model has a built-in Monte Carlo population simulator, referred to as HTTK-POP (Ring et al., 2017), which can account for inter-individual variability in the human population. HTTK-POP uses physiological metrics, based on different demographics and subgroups from National Health and Nutrition Examination Survey (NHANES) data (Johnson et al., 2014). These include gender, age, body weight class, renal function, and ethnicity. HTTK-POP varies several parameters, each with a coefficient of variation of 30%, including liver volume, cell density, blood flow, body weight, glomerular filtration rate, and intrinsic hepatic clearance (Cl_int_). The default setting of 1000 simulations was used to provide a C_ss_ distribution, and the 95^th^ percentile was used to derive the AED. Thus, the AED that was reported as POD_Bioactivity_ for each chemical was obtained by dividing the 5^th^ percentile AC_50_ concentration by the 95^th^ percentile C_ss_ from a constant dose rate of 1 mg/kg per day. To model each C_ss_, the calc_mc_css() function in HTTK was used with output.units=”uM” and well.stirred.correction=TRUE.

### High-throughput toxicokinetics gap-filling

To run the 3compartmentss model, specific *in vitro* parameters and physical chemical properties are required. Specifically, the requirements to run the model and return units in mg/kg bw/day are Cl_int_, fraction unbound in the plasma protein (F_up_), molecular weight, and the octanol/water partition coefficient (log P). These data are available in HTTK for many DSL chemicals but are unavailable for thousands of others (Fig. 1), and therefore, *in silico* predictions were used to address this data gap. The ChemmineOB R package^7^ (version 1), which interfaces the OpenBabel C++ project (O’Boyle et al., 2011), was used to provide molecular weight and log P values for each chemical as required by the HTTK model. ADMET Predictor 10 used the simplified molecular-input line-entry system (SMILES) of each chemical to predict F_up_ percentage (hum_fup%) and human liver microsomal clearance (CYP_HLM_Cl_int_). Recent work has demonstrated that ADMET Predictor estimates of F_up_ are reliable and estimates of intrinsic clearance (Cl_int_) are adequate, allowing for the calculation of stable C_ss_ values within the applicability domain of the model (Pradeep et al., 2020). ADMET parameters were formatted to HTTK units following a previously applied procedure (Rajkumar et al., 2021). Hum_fup% were converted to F_up_ by dividing by 100. CYP_HLM_Cl_int_ (μL/min/mg) were adjusted to Cl_int_ HTTK units (μL/min/10^6^ cells) by dimensional analysis using scaling factors (Barter et al., 2007) that have been previously applied (Sipes et al., 2017):

(Eq. 2) $$Cl_{int}=CYP\text{\_}HLM\text{\_}CL_{int}\times \frac{32\;mg\;of\;microsomal\;protein}{g\;of\;liver}\times \frac{1\;g\;of\;liver}{99\;\times \;10^{6}\;cells}$$

ADMET Predictor 10 was also used to estimate fraction absorbed and fraction bioavailable. These parameters were not used in the HTTK model but were used to filter the HTTK data. Specifically, chemicals with a fraction absorbed or bioavailable below 0.1 were filtered out, as these chemicals are predicted to have a fraction absorbed or bioavailability that is one order of magnitude away from the assumption of full absorption/bioavailability made by the model. DSL chemicals outside the applicability domain of any of the predictions made by ADMET Predictor 10 were noted and filtered out. Lastly, the Lipinski rule of five (Lipinski et al., 1997) was used to exclude chemicals with more than 5 hydrogen bond donors, more than 10 hydrogen bond acceptors, molecular weight above 500 Da, and a log P above 5. The Lipinski rule of five filter was applied to minimize uncertainty around the *in silico* predictions, as these models were trained using mainly pharmaceutical data. The rule of five violations were identified using the R Chemistry Development Kit (rcdk) library, based on the open-source cdk Java library (Steinbeck et al., 2003).

In order to build confidence in the *in silico* toxicokinetics parameters, ADMET Predictor 10 was first applied to 931 chemicals in HTTK (Fig. 2). A C_ss_ for each of the chemicals was obtained using the existing HTTK data. Subsequently, F_up_ and Cl_int_ values derived from ADMET Predictor 10 were incorporated into HTTK using the add_chemtable( ) function with over-write=TRUE. For each chemical, an *in silico*-derived C_ss_ was then modeled and compared against the *in vitro*-derived C_ss_.

The workflow was applied to unique DSL chemicals containing structural information (i.e., SMILES). Specifically, the required data were obtained from ADMET Predictor 10 and ChemmineOB and then incorporated into HTTK using add_chemtable() with overwrite=FALSE. Setting overwrite to FALSE prioritized using existing experimental HTTK data over the provided *in silico* data where available. To be conservative, a cut-off C_ss_ value of 0.1 μM was applied (i.e., C_ss_ values below 0.1 defaulted to 0.1), as there are only 12 chemicals in HTTK with a C_ss_ based on *in vitro* data below 0.1.

### Generalized read-across using molecular fingerprints

GenRA (Helman et al., 2019; Shah et al., 2016), an algorithmic approach to read-across that has been previously developed and implemented within the EPA’s CompTox Chemicals Dashboard, was explored as a means to predict Tox-Cast bioactivity data outcomes for DSL chemicals lacking experimental data. Specifically, structurally similar analogues were identified from the Tox-Cast database on the basis of different chemical fingerprints. Pairwise similarity was calculated using Tanimoto coefficients. Similarity scores were based on molecular fingerprint similarities between chemicals. Three different chemical fingerprints were explored to optimize the read-across approach: ToxPrint, PubChem, and Morgan fingerprints. The protocol to calculate ToxPrint fingerprints involves multiple steps within and outside the R workflow. First, SMILES for ToxCast and DSL chemicals were converted to structure-data files (SDFs) using the ChemminerR package (Cao et al., 2008). The SDFs were imported into the Chemotyper v1.0.r12976 software (Yang et al., 2015) and converted to ToxPrints using the ToxPrintv2.0_r711.xml template (done outside of the R workflow environment). A fingerprint file was exported from the Chemotyper and imported into the R workflow. PubChem and Morgan fingerprints were calculated using the rcdk library.

GenRA uses similarity-weighted activity values of analogs to automate read-across predictions of biological activity for data-poor target chemicals (Shah et al., 2016). We applied the GenRA algorithm to estimate *in vitro* bioactivity concentrations for chemicals lacking ToxCast data using the following equation:

(Eq. 3) $$Bioactivity_{Read-Across}=\frac{\sum_{i}^{k}S_{i}Bioactivity_{i}}{\sum_{i}^{k}S_{i}}$$

where Bioactivity_Read-Across_ is the estimated log_10_bioactivity concentration using GenRA, S_i_ is the Tanimoto coefficient of the analog, Bioactivity_i_ is the log-transformed 5^th^ percentile bioactivity concentration of the analog, and k is the number of nearest neighbors. The k-value was set to 10 as done previously (Helman et al., 2019), but different s-values ranging from 0.1 to 0.8, in 0.1 increments, were explored to optimize the performance relative to coverage.

In an effort to establish confidence in the read-across approach, ToxCast chemicals were used as a control. Each ToxCast chemical (target) was iteratively compared to the other ToxCast chemicals. The ten nearest neighbors (analogues) with the highest Tanimoto coefficients above the threshold s-value were identified for each ToxCast chemical. To qualify as a target or analogue, the chemical required more than five active assays and more than five active structural features (fingerprint bits). The 5^th^ percentile bioactivity concentrations for the structurally similar chemicals were reported, and the GenRA equation was used to predict a bioactivity concentration for the target chemical. The bioactivity concentration for each chemical derived from ToxCast data was compared to the bioactivity concentrations derived from read-across to assess the performance of the read-across approach. The fingerprint type and s-value combination that returned the optimal number of targets and accuracy was identified for further application to DSL chemicals.

Following the same protocol, the DSL chemicals were iteratively compared to all the ToxCast chemicals to identify structurally similar chemicals. The GenRA equation was used to predict bioactivity for DSL chemicals with more than five active structural features based on the ten or fewer analogues above the optimal s-value. IVIVE was then applied to derive AEDs for these chemicals. The POD_Read-Across_ was calculated by dividing the GenRA-predicted concentration by the C_ss_ of the target. Up to ten additional AEDs were calculated for each target by dividing the 5^th^ percentile ToxCast bioactivity concentration of the analogues by the C_ss_ of the target.

### Collection of POD_Traditional_ data

Only published *in vivo* data were used as part of this work, and no new animal studies were conducted. For the chemicals where a POD_Bioactivity_ or POD_Read-Across_ could be derived, POD_Traditional_ data were downloaded as available from ToxValDB (latest version as of September 17, 2020) hosted on the EPA CompTox Chemicals Dashboard^3^ (Williams et al., 2017). Data were filtered to only include POD_Traditional_ values where the units could be reported as mg/kg or mg/kg-day. Only the most common response types (LOAEL, NOAEL, BMDL) were retained, and synonyms of these response types were converted accordingly. Specifically, “NOEC”, “NOAEC”, “NOEL”, “NEL”, “HNEL” were labelled as “NOAEL”, and “LOEC”, “LOAEC”, “LOEL”, “LEL” were labelled as “LOAEL.” Exposure route was limited to oral and gavage routes. Risk assessment class and study type were limited to developmental, reproductive, subchronic, chronic, and repeat dose. The lowest POD in ToxValDB for each chemical was used as the POD_Traditional_.

### Collection of exposure estimates data

Exposure data were downloaded from the CompTox Chemicals Dashboard (Williams et al., 2017). Specifically, the Chemical Abstracts Service Registry Number (CASRN) for each DSL chemical was input into the dashboard and the NHANES/Predicted Exposure data were downloaded on August 13, 2020. The SEEM3 ExpoCast median and 95^th^ percentile values were used as the denominators in the BER calculations, with the 95^th^ percentile exposure estimates providing the more conservative BERs.

### Determination of threshold of toxicological concern values

The SDF file for the DSL chemicals, described previously, was loaded into KNIME (v 4.2.1), and the RDKit salt stripper node was used to convert organic chemicals with counter ions to their neutral form. The converted SMILES were then loaded into Tox-tree (v3.1.1) software (Patlewicz et al., 2008), and the Cramer class was assigned for each DSL chemical in batch mode. Cramer classes were limited to Class I (TTC: 30 μg/kg bw/day), Class II (TTC: 9 μg/kg bw/day), and Class III (TTC: 1.5 μg/kg bw/day) (EFSA and WHO, 2016), as genotoxicity is beyond the scope of this work.

## 3. Results

### 3.1 Intersection between domestic substances list and data sources

Data extraction from ToxCast resulted in AC_50_ concentrations for a total of 8,059 chemicals. For 128 of the chemicals, there were no active assays after filtering, and AC_50_ concentrations were assigned as 100 μM. The version of HTTK used in the workflow had sufficient *in vitro* data to model C_ss_ for 931 chemicals. Cross-referencing the DSL CASRN for 17,095 unique chemicals against the CASRN from ToxCast and HTTK revealed that there was sufficient data to model bioactivity-derived PODs for 357 chemicals (Fig. 1). Two main data gaps existed for the remaining chemicals. For the inner data gap, there were AC_50_ concentrations for 2,625 DSL chemicals but no HTTK data. In the outer gap, there were 14,096 chemicals that had neither HTTK nor ToxCast data. There were 17 DSL chemicals that had HTTK data but no ToxCast data available.

### 3.2 Addressing the HTTK data gap

#### 3.2.1 Building confidence for *in silico* predictions

In total, *in silico* F_up_ and Cl_int_ values were obtained for 931 chemicals in the HTTK R package. The *in silico* F_up_ values ranged from 0.018 to 0.998, the upper range being equivalent to the chemical being freely available. In HTTK, the F_up_ ranged from 0 to 1, with the limit of detection defaulting to 0.005 when F_up_ = 0. The *in silico* Cl_int_ values ranged from 0.909 to 4848.485 μL/min/10^6^, and the HTTK values ranged from 0 to 1000 μL/min/10^6^. Thus, the *in silico* Cl_int_ values had a higher maximum clearance than experimental measurements.

**Fig. 3:**
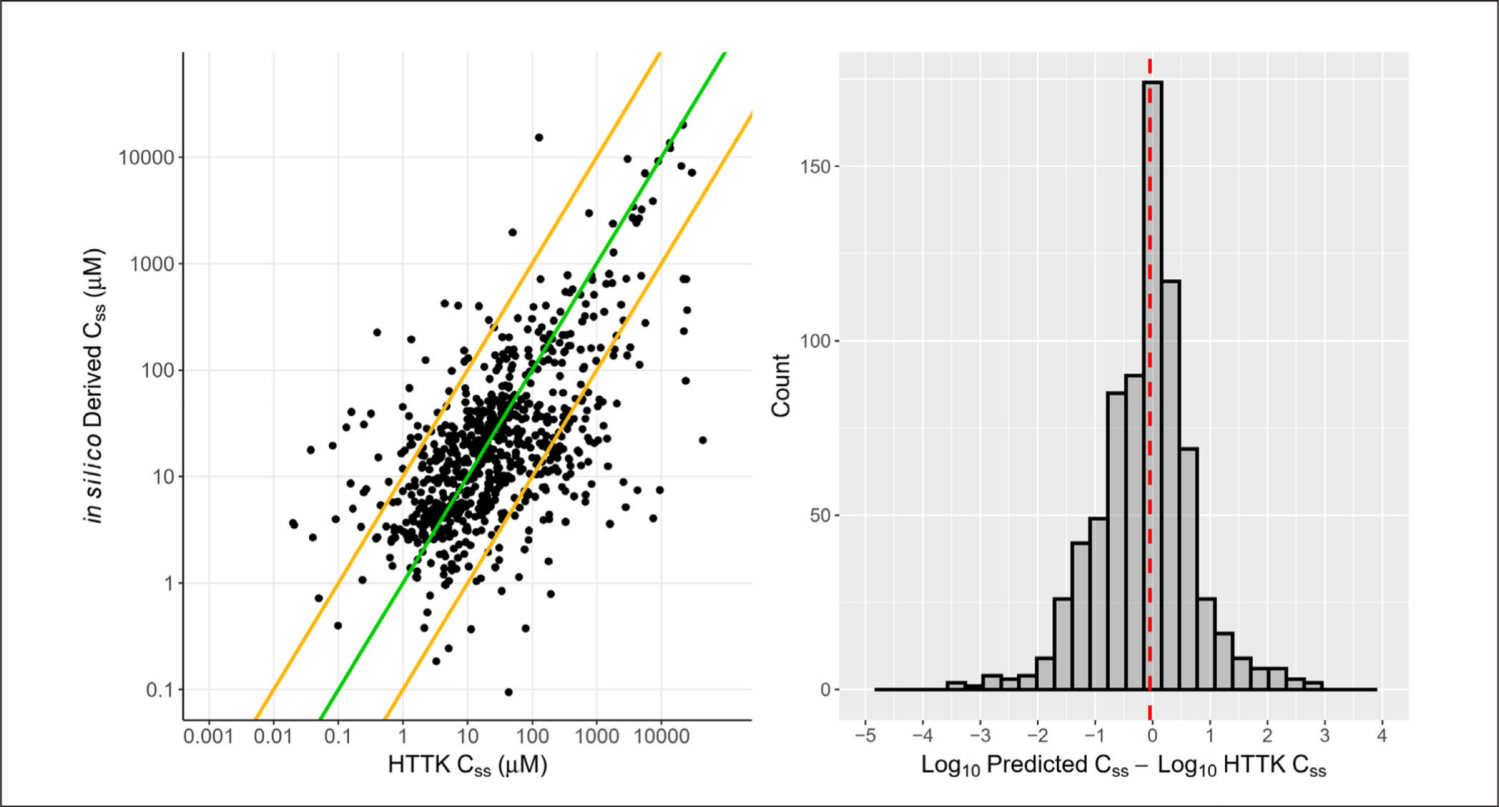
Comparison of C_ss_ derived from *in silico* parameters with C_ss_ derived from *in vitro* parameters Left scatterplot displays correlation of *in silico*-derived C_ss_ and HTTK C_ss_ derived from *in vitro* parameters. Green line represents perfect correlation and orange lines display boundary where C_ss_ values are within 10-fold of each other. Right histogram shows the distribution of log_10_C_ss_ ratios between predicted (*in silico*) and HTTK (*in vitro*). Deriving C_ss_ values using *in silico* parameters revealed that 79.68% of C_ss_ values derived from *in silico* predictions are within 10-fold of the C_ss_ derived using HTTK data, and 96.64% are within 100-fold (adjusted r^2^ = 0.3624).

Without applying filters, deriving C_ss_ values using *in silico* parameters revealed that 75.94% of C_ss_ values derived from *in silico* predictions were within 10-fold of the C_ss_ derived using HTTK data, and 94.31% were within 100-fold^8^. Applying the filters outlined below removed 188 chemicals and improved the accuracy with 79.68% of predictions being within 10-fold of HTTK C_ss_ and 96.64% within 100-fold (Fig. 3; Tab. S1^8^). The *in silico*-derived C_ss_ values, after filtering, were more often lower than the *in vitro* derived value (less conservative), with 401 *in silico* estimations resulting in a lower C_ss_ compared to 342 estimates with a higher C_ss_.

The Lipinski rule of five filter removed the most chemicals (119 removed) with 94 of those chemicals being unique to this filter alone (Fig. S1^8^). 31 unique chemicals were removed by the applicability domain filter. All of the chemicals removed by the fraction absorbed filter were also removed by the fraction bioavailable filter, making the former filter redundant in this application. Together, the fraction absorbed and bioavailable filters removed 37 unique chemicals, with the latter removing an additional 5 unique chemicals.

The discrepancies between *in silico*-derived C_ss_ and *in vitro*-derived C_ss_ ranged from −6.44 to 6.52 on the log scale (log_10_*in silico*-derived C_ss_ - log_10_ *in vitro*-derived C_ss_ ) without filtering. After applying filters, the range narrowed to −3.30 to 2.75 (Tab. S2^8^). There were only 11 cases where the *in silico*-derived C_ss_ was between 100- and 1,000-fold lower than the *in vitro*-derived C_ss_, and 11 cases where the *in silico*-derived C_ss_ was between 100- and 1,000-fold higher than the *in vitro*-derived C_ss_. For the largest discrepancies, there were three instances where the *in silico*-derived C_ss_ was > 1,000-fold higher than the *in vitro*-derived C_ss_ (cotinine, 4-chloro-2-methylaniline, and chlorophene).

Some of the largest discrepancies between C_ss_ values pre-filtering were associated with specific structural congeners. Specifically, the chemicals that had *in silico* C_ss_ more than 100,000 lower than the *in vitro* C_ss_ were enriched with ToxPrint chemotypes related to aromatic halides. However, this result was not significant after adjusting for multiple comparisons (Holm-adjusted Fisher’s Exact test). Five of the eight chemicals with discrepancies larger than 100,000-fold were PCBs. One additional chemical related to PCBs, p,p’-DDD, also had a largely discrepant C_ss_. The *in vitro* TK data for the PCBs comes from Tonnelier et al. (2012), and the data for p, p’-DDD comes from Wetmore (2015). The F_up_ defaulted to 0.005 for the PCBs and was 0.03 for p, p’-DDD. The *in silico* F_up_ predictions for these chemicals were similar to the *in vitro* measurement of p, p’-DDD around 0.03. The *in vitro* Cl_int_ value for these chemicals ranged from 2.70 × 10^−4^ to 0 μL/min/10^6^ cells. In contrast, the Cl_int_ predictions were 2180.8 for p,p’-DDD and the maximum value of 4848.5 μL/min/10^6^ cells for the PCBs. Thus, the differences in C_ss_ are attributed to the vastly different Cl_int_ values. All of these chemicals were removed from analysis when the filters were applied. There did not appear to be any functional groups associated with *in silico* C_ss_ values that are higher than the *in vitro* C_ss_ before or after filtering.

#### 3.2.2 Applying *in silico* HTTK data to DSL chemicals

**Fig. 4:**
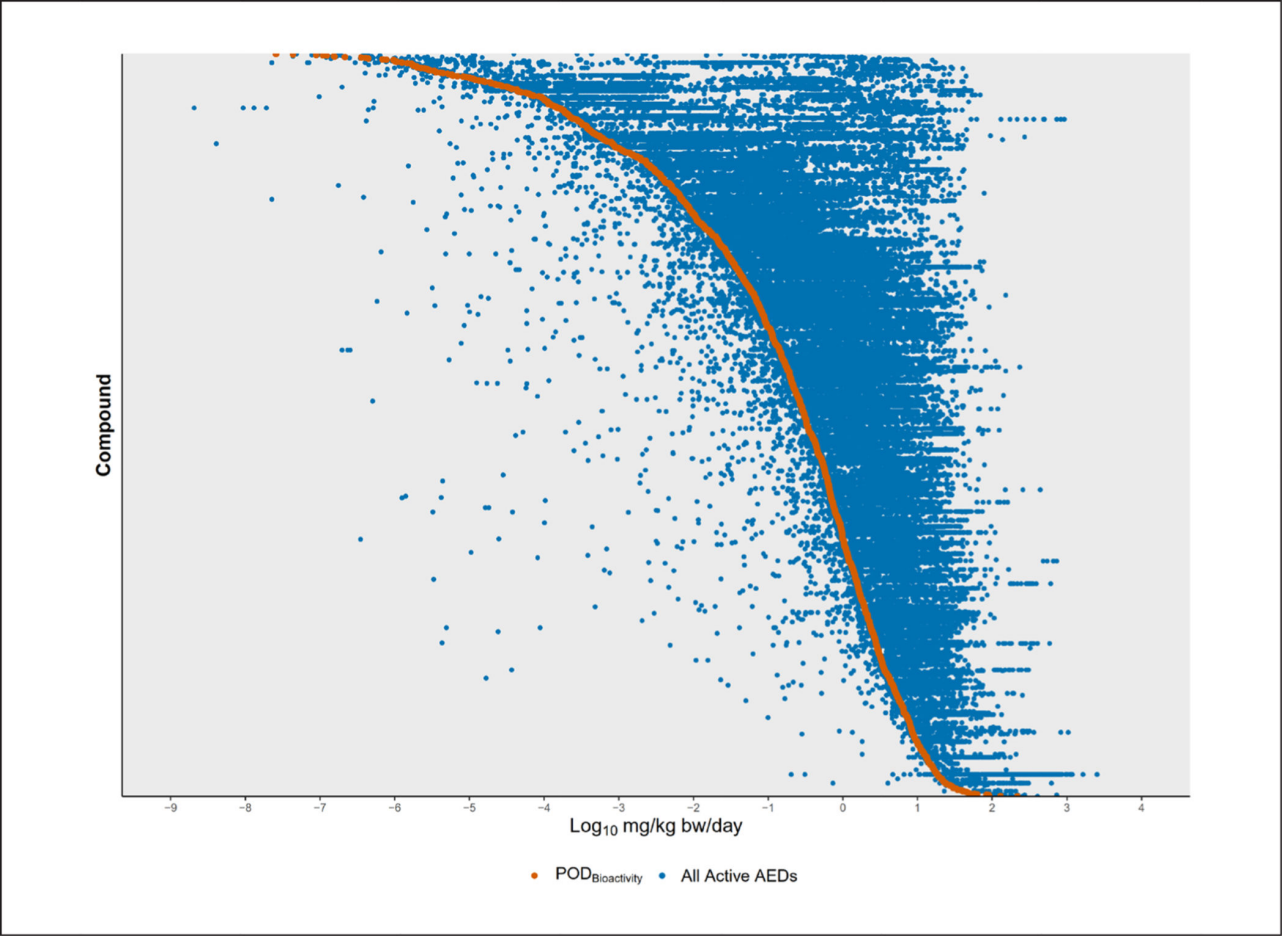
Ranking of DSL chemicals present in ToxCast by POD_Bioactivity_ POD_Bioactivity_ values (orange) could be derived for 1,708 chemicals after addressing the HTTK data gap and applying filters. Blue circles indicate all the AEDs based on each individual ToxCast assay result used to derive the POD_Bioactivity_.

The requisite data to run the 3compartmentss model was available for 16,637 DSL chemicals. From these chemicals, POD_Bioactivity_ values could be derived for a total of 2,974 chemicals. All of the previous filters were applied, resulting in the removal of 1,266 POD_Bioactivity_ values and leaving a POD_Bioactivity_ for 1,708 DSL chemicals (Fig. 4). The rest of the DSL chemicals lacked ToxCast bioactivity data, and a POD_Bioactivity_ could not be derived. Across all 16,637 DSL chemicals, the C_ss_ concentrations ranged from 0.1 μM (default minimum) to 28924.64 μM. A total of 2,127 DSL chemicals had the minimum C_ss_.

### 3.3 Addressing the ToxCast bioavailability data gap

#### 3.3.1 Optimization of generalized read-across using ToxCast bioactivity data

GenRA was explored using Morgan, PubChem, and ToxPrint fingerprints. ToxCast chemicals were retained if they had more than five active assays and more than five active fingerprint features (bits). 4,934 chemicals passed this criterion for Morgan fingerprints, 4,945 chemicals for PubChem fingerprints, and 4,369 chemicals for ToxPrint chemotypes. The number of targets where read-across could be applied increased as the s-value was relaxed. Read-across could be applied to all targets, for each fingerprint type, when the s-value reached 0.1 (Fig. S2^8^). Although ToxPrint chemotypes allowed fewer possible targets and analogues to be used in read-across, ToxPrint served as the most accurate fingerprint type for read-across (Fig. S3, S4^8^). Specifically, an s-value of 0.3 gave a read-across concentration that was within 10-fold of the true bioactivity concentration for 63.99% of chemicals and within 100-fold for 89.17% of chemicals (Fig. S5^8^). The possible bioactivity concentrations ranged from 8.81 × 10^−7^ to 342 μM on the arithmetic scale (8.6 orders of magnitude). Thus, the accuracy was not a result of the dynamic range of possible bioactivity concentrations. Considering that ToxPrint chemotypes are a fixed set, interpretable, and were developed with a stronger focus on mechanistic modes of action and a higher relevance to toxicological effects (Richard et al., 2016; Yang et al., 2015), these fingerprints and an s-value of 0.3 were chosen as the optimal parameters to perform GenRA on DSL chemicals.

#### 3.3.2 Assessing the accuracy of *in silico* HTTK data combined with generalized read-across

**Fig. 5:**
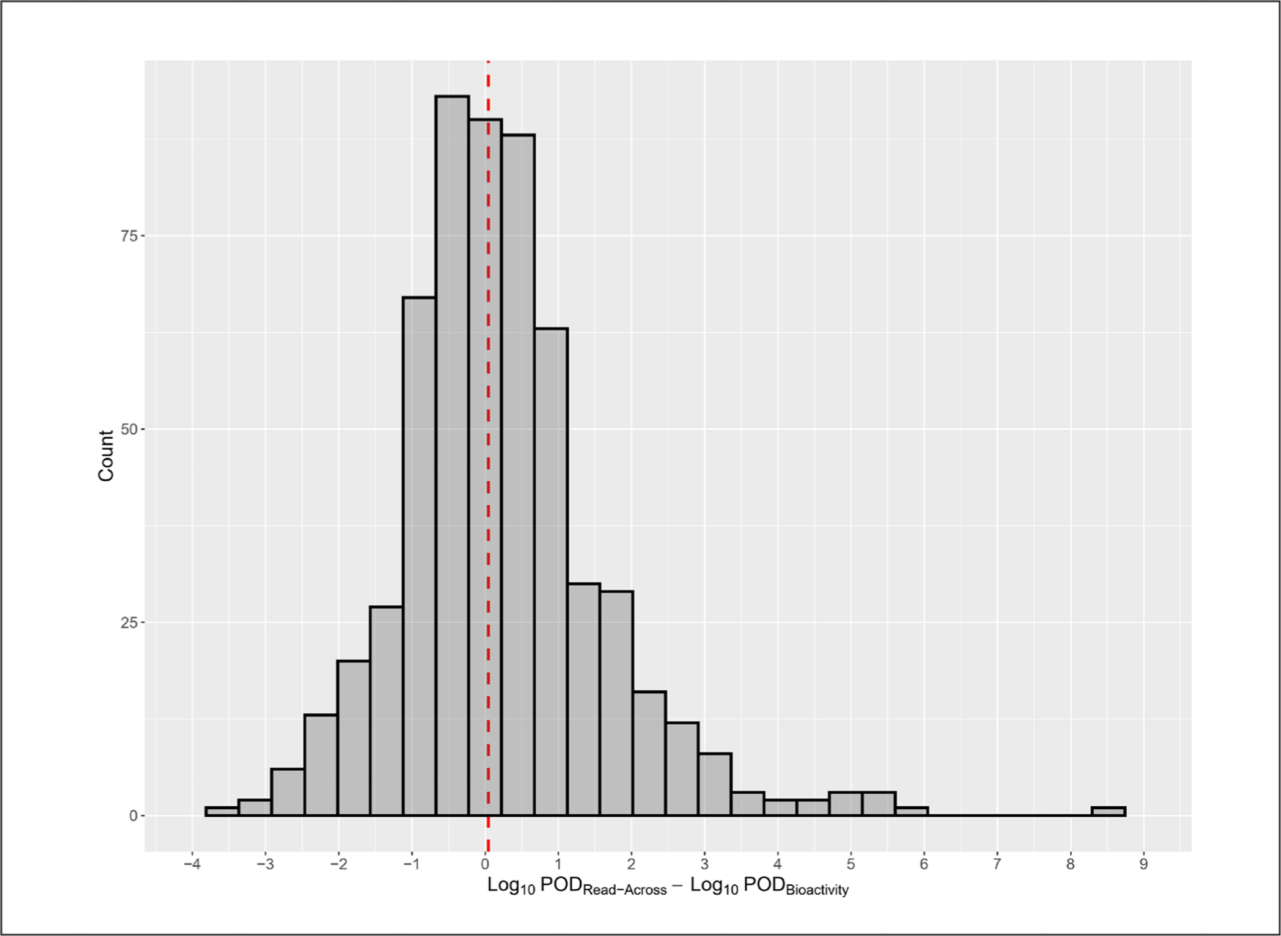
Comparison of POD_Read-Across_ with true POD_Bioactivity_ derived from ToxCast and *in vitro* HTTK data The majority of chemicals (91.21%) have a POD_Read-Across_ within 100-fold of the true POD_Bioactivity_ (adjusted r^2^ = 0.1955).

To test the effects of compounding uncertainty with *in silico* HTTK data and read-across bioactivity concentrations, the POD_Read-Across_ was compared to the true POD_Bioactivity_ from ToxCast, where possible. The true POD_Bioactivity_ was calculated by dividing the 5^th^ percentile bioactivity concentration for each chemical by the *in vitro*-derived C_ss_, while the POD_Read-Across_ was calculated by taking the read-across bioactivity concentration, calculated by the GenRA equation, and dividing it by the *in silico*-derived C_ss_. There were 580 chemicals for which comparisons could be made with HTTK filters applied and 733 chemicals without the application of filters. The filtered data demonstrated that the POD_Read-Across_ was within 10-fold of the true POD_Bioactivity_ for 79.48% of chemicals, and within 100-fold for 91.21% of chemicals (Fig. 5). The possible POD_Bioactivity_ values ranged from 1.57 × 10^−9^ to 246 on the arithmetic scale (11.2 orders of magnitude). Thus, the accuracy was not a result of the dynamic range of possible POD_Bioactivity_ values. Interestingly, the POD_Read-Across_ was a better surrogate of POD_Bioactivity_ than the read-across concentration was for the bioactivity concentration alone. Thus, there do not appear to be any issues related to uncertainty propagation.

#### 3.3.3 Applying generalized read-across to DSL chemicals

**Fig. 6:**
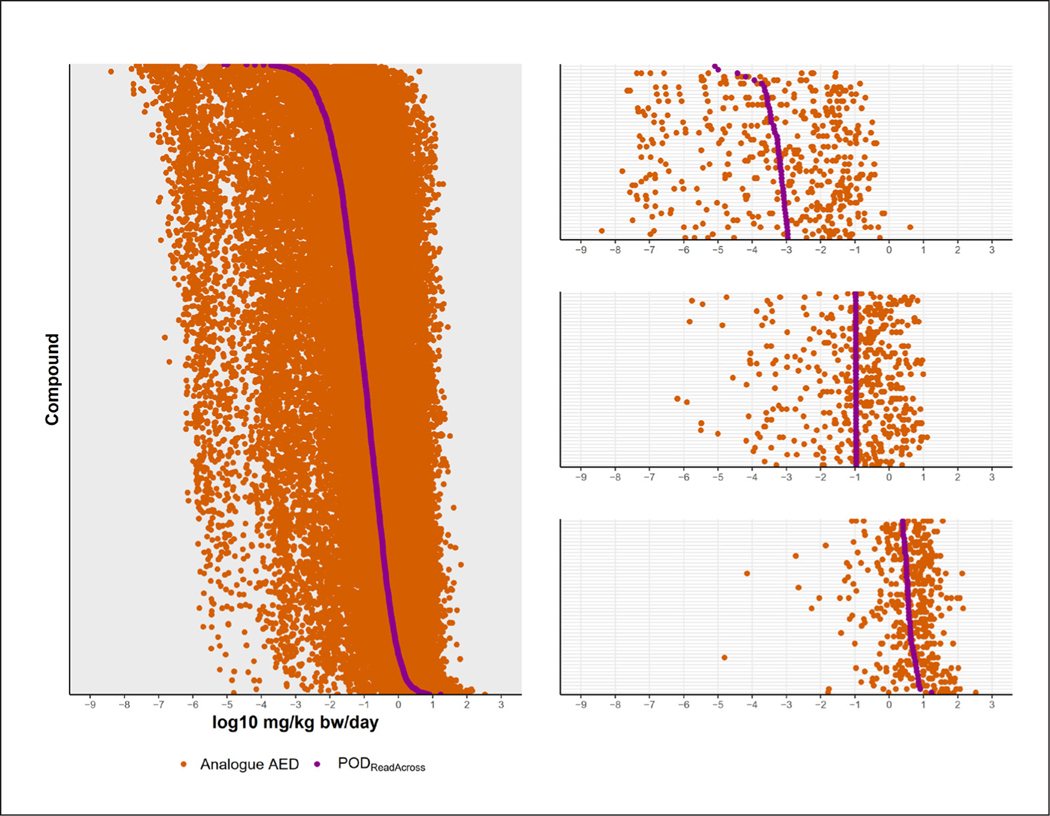
Ranking of DSL chemicals absent in ToxCast by POD_Read-Across_ PODs could be derived for 4,093 DSL chemicals using GenRA and *in silico* HTTK parameters. Each row presents the POD_Read-Across_ derived from read-across (purple) and the analogue AEDs used in the derivation (orange; AEDs are calculated using the ToxCast bioactivity concentration of the analogue divided by the C_ss_ for the target). The top panel on the right shows the 50 chemicals with the lowest POD_Read-Across_ values, middle panel shows the 50 chemicals around the median POD_Read-Across_, and the bottom panel shows the 50 chemicals with the highest POD_Read-Across_ values.

Using the same read-across protocol as above (> 5 active ToxPrint chemotypes; s-value of 0.3), a POD_Read-Across_ could be predicted for 9,937 DSL chemicals due to the overlap in structural features between many ToxCast and DSL chemicals (Fig. S6^8^). After applying HTTK filters, 4,093 chemicals remained with a POD_Read-Across_ (Fig. 6). In total, there were 12,828 DSL chemicals with a derived POD based on ToxCast bioactivity (2,974) or read-across (9,854). After filtering, there were 5,801 chemicals with a POD based on ToxCast (1,708) or read-across (4,093). The log_10_PODs ranged from −7.59 to 2.34 for the DSL chemicals passing filtering criteria.

### 3.4 Comparison of bioactivity PODs to traditional PODs

**Fig. 7.**
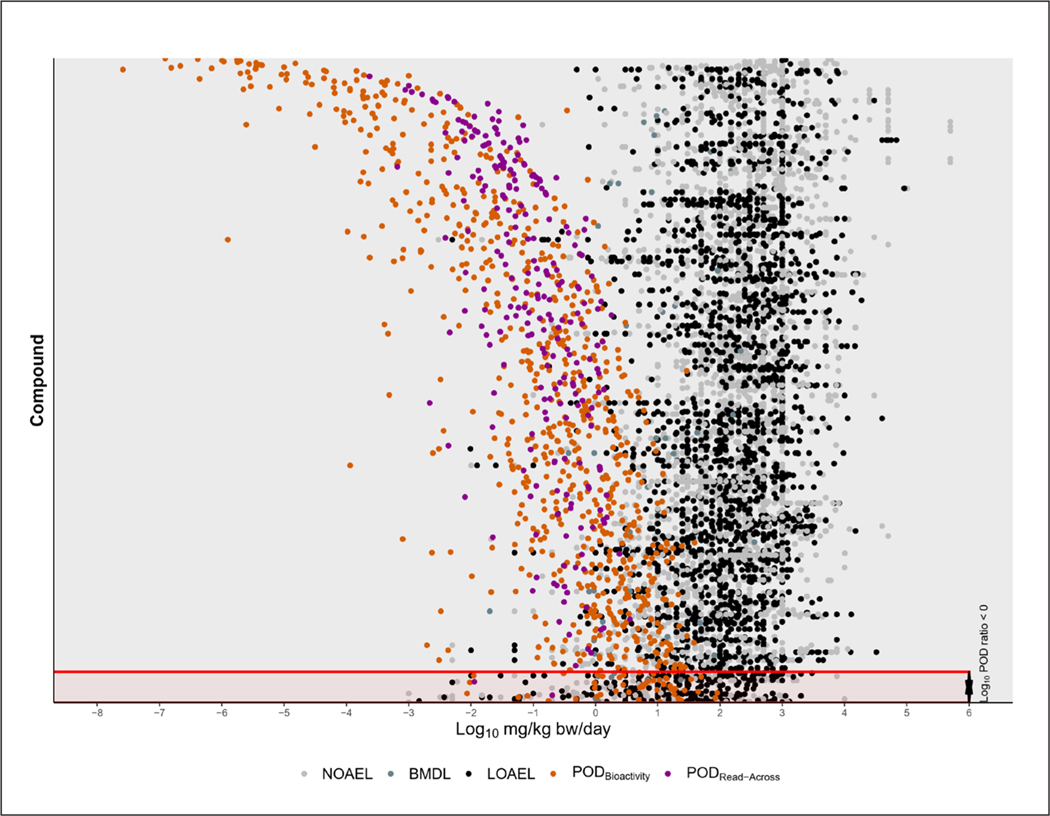
Comparison between POD_Bioactivity_ and POD_Read-Across_ with POD_Traditional_ Each line represents a chemical with the POD_Bioactivity_ in orange or POD_Read-Across_ in purple, while the POD_Traditional_ values are represented in grayscale. Chemicals are ordered by the POD ratio (log_10_POD_Traditional_ - log10POD_Bioactivity_ or log_10_POD_Traditional_ - log_10_POD_Read-Across_). Chemicals for which the POD_Bioactivity_ or POD_Read-Across_ values were not protective are highlighted in red at the bottom. The CASRN and structures for these chemicals are available^7^.

Among the chemicals with a POD_Bioactivity_ or POD_Read-Across_, a total of 2,248 chemicals had a suitable POD_Traditional_ in Tox-ValDB with a response type of NOAEL, BMDL, or LOAEL (Tab. S3^8^). After applying the HTTK filter, 1,042 comparisons could be made. The vast majority of chemicals (95.20%) had POD_Bioactivity_ or POD_Read-Across_ values that were protective (Fig. 7), in that they were lower than or equal to POD_Traditional_ (see Tab. S3^8^ for more detailed comparisons). The median difference between POD_Traditional_ and POD_Bioactivity_ or POD_Read-Across_ was 241-fold on an arithmetic scale, indicating that on average the POD_Bioactivity_ or POD_Read-Across_ is two orders of magnitude lower than POD_Traditional_. The POD_Bioactivity_ or POD_Read-Across_ values were least protective when compared to BMDL, with five of the 42 PODs not being protective of BMDL (11.90%). Analysis of the ToxPrint chemotypes of chemicals without a protective POD revealed an enrichment of four chemotypes: bond:metal_group_III_other_Sn_generic, atom:element_metal_poor_metal, bond:X[any]_halide, and bond:CS_sulfide (Holm-adjusted Fisher’s Exact p-value < 0.01). After applying HTTK filters, there were no enriched chemotypes for chemicals with non-protective PODs, demonstrating the utility of applying filters to obtain protective PODs.

In the APCRA case study, there were some ToxPrint chemotypes that were enriched in chemicals with non-protective POD_Bioactivity_ values. In this analysis, only one of these chemotypes (bond:CS_sulfide) was enriched in chemicals with non-protective POD_Bioactivity_ or POD_Read-Across_ values, but the result was not significant after adjusting for multiple comparisons (Holm-adjusted Fisher’s Exact test). This may be because these structural features are underrepresented in the DSL. For example, the chemotype bond:P=O_phosphate_thio was not present in any DSL chemicals analyzed.

### 3.5 Derivation of bioactivity exposure ratios

**Fig. 8:**
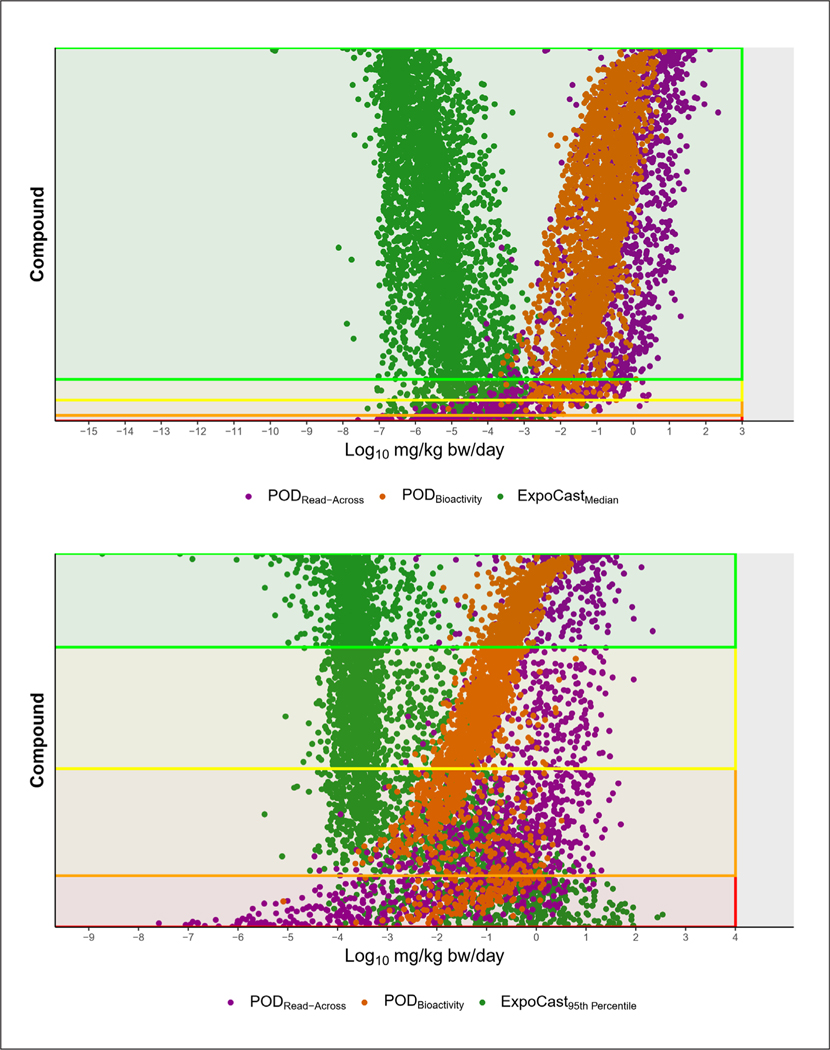
Bioactivity exposure ratios Exposure estimates were based on the ExpoCast median value (green) and compared against the POD_Bioactivity_ (orange) and POD_Read-Across_ (purple). The top panel displays BERs based on ExpoCast median exposure predictions, and the lower panel displays BERs based on the 95^th^ percentile prediction. Red shaded areas indicate log_10_BER < 0, orange shaded areas display log_10_BER 0–2, yellow shaded areas display log10BER 2–3, and green shaded areas indicate log_10_BER > 3.

Exposure estimates were available to generate 7,042 BERs and of these 3,680 were retained after applying the filters (Fig. 8). The BERs were separated into bins of variable levels of potential risk: log_10_BER < 0, log_10_BER 0–2, log_10_BER 2–3, log_10_BER > 3. The first and second bins contain chemicals with the highest potential for concern, as the POD_Bioactivity_ values are below or approaching the exposure estimate. Previous work has shown that these bins capture chemicals previously assessed and concluded to be toxic to human health or the environment under Section 64 of the Canadian Environmental Protection Act (CEPA), 1999 (Health Canada, 2021). When the ExpoCast median exposure predictions are used to derive BERs, the results show that there are 55 chemicals with a log_10_BER < 0 and 149 chemicals with a log_10_BER 0–2. Furthermore, there are 206 chemicals with a log_10_BER 2–3 that may be considered on a case-by-case basis, and 3,270 chemicals with a log_10_BER > 3. Using the Expo-Cast 95^th^ percentile exposure prediction increases the number of chemicals to 505 in the log_10_BER < 0 bin, 1,054 in the log_10_BER 0–2 bin, and 1,200 in the log_10_BER 2–3 bin. The remaining 921 chemicals had a BER > 3.

### 3.6 Comparison of TTC values with bioactivity exposure ratios

The TTC and BER approaches can be seen as complementary to each other, as both might be used to assist in prioritization efforts. Thus, the POD_Bioactivity_ and POD_Read-Across_ values were compared to the TTC values to see how they might support each other. As was demonstrated in the APCRA case study, the TTC was found to be lower than the POD_Bioactivity_ or POD_Read-Across_ for the majority of chemicals (88%; Fig. S7^8^). On the arithmetic scale, the median difference showed that the TTC was on average 25 times lower than the bioactivity PODs. As a further comparison, the chemicals where the exposure estimate was greater than the TTC were compared against the chemicals with a log_10_BER < 0 or log_10_BER of 0–2 (Fig. S8^8^). This exercise determined that 422 chemicals with a log_10_BER < 0 and 489 chemicals with a log_10_BER of 0–2 also had a TTC that was below the exposure estimate. Thus, these are chemicals with multiple lines of evidence supporting higher potential for concern and are candidates that may therefore warrant closer evaluation. There were 243 chemicals with a TTC that was below the exposure estimate that were not in the high-concern BER bins. For these chemicals, expert judgement could be applied to determine whether these chemicals should be further evaluated in subsequent scoping steps of a screening approach.

## 4 Discussion

In this work, we presented a computational workflow developed to begin to address data gaps for a broad chemical space as represented by the Canadian DSL. Specifically, we applied *in silico* tools and read-across to derive PODs for DSL chemicals based on bioactivity data from qHTS programs. The intended purpose of this workflow is to identify data-poor chemicals with the highest potential for concern that, with additional scoping as needed, may be candidates of interest for further prioritization and assessment activities. This analysis serves as a direct follow-up to the collaborative retrospective case study that demonstrated the utility of these *in vitro* bioactivity data to derive protective PODs and BERs to be used to support chemical risk prioritization (Paul Friedman et al., 2019). In the retrospective case study, the analysis was applied to 448 chemicals and was the largest analysis hitherto. Herein, we expanded on this work and applied the methodology to 12,828 chemicals with a derived POD based on ToxCast bioactivity or read-across, of which 3,679 had physico-chemical properties amenable to HTTK modeling and exposure estimates available for BER derivation. Further advancements to the approach, such as the inclusion of other data sources and addressing areas of uncertainty, will serve to broaden the scope of application to include more diverse chemicals represented in chemical inventories.

Given that the primary application context of qHTS data and the BER approach is to serve as a risk-based screening tool in prioritization activities (Thomas et al., 2013a), the various decisions related to the derivation of the PODs and BERs were made to be conservative to address the different areas of uncertainty. Consequently, the POD_Bioactivity_ or POD_Read-Across_ were found to be lower than the POD_Traditional_ for 95% of chemicals. However, these decisions may have reduced the correlation between the qHTS-based POD_Bioactivity_ and animal-based POD_Traditional_ for the chemical space evaluated as demonstrated previously (Wignall et al., 2018). The use of POD_Traditional_ values derived mainly from rodent studies, often using a limited dose range and few biological endpoints with limited mechanistic information, presents a challenge for building confidence in our workflow. This is because the POD_Bioactivity_ values were based on a broad concentration range to measure high-precision AC_50_ values, a large number of toxicological endpoints probing all of known biology, primarily assays using human cells, and a toxicokinetics model simulating chemical disposition in humans. Furthermore, the type of toxicity value available from traditional data also makes the comparisons difficult. For example, there were relatively few BMDL values available in ToxValDB for DSL chemicals. Interestingly, the POD_Bioactivity_ values were least protective relative to BMDLs, potentially due to the BMDL values being more reflective of true *in vivo* bioactivity compared to the other toxicity values. The purpose of this approach is not to predict a POD to serve as a replacement for animal data in a quantitative risk assessment. Rather, this approach is meant to identify chemicals with a higher potential for concern and support a weight-of-evidence assessment. The benefit of the qHTS data is that it provides mechanistic information for known biology and adverse outcomes. Chemicals with high hazard (low PODs) or high risk potential (low BERs) are prioritized for further examination, and the lowest active assays for these chemicals, or analogues in the case of GenRA, can be used to inform where more focus is needed in the evaluation. This would reduce the need for unnecessary toxicity testing, providing a need for only the most targeted or relevant studies serving to greatly reduce the number of animals required to inform a chemical safety evaluation. There are some areas of uncertainty that remain inherent in the methodology and acknowledging these can focus future research efforts to improve the approach and support the transition away from animal use in toxicity assessment.

Some of the uncertainties revolve around the completeness of the toxicological space covered by the test batteries used to calculate bioactivity. ToxCast consists of nearly 1,400 assays (Richard et al., 2016), covering a broad range of possible adverse outcomes, but this is still likely insufficient to accurately capture the potencies of all possible biological effects, and not all of the nearly 1,400 assays are tested for each chemical. For example, it is acknowledged that chemicals with structural features related to carbamates or organophosphates are not adequately addressed by ToxCast (Paul Friedman et al., 2019). Specifically, these chemicals and their metabolites are potent acetylcholinesterase inhibitors, and while there are assays that measure acetylcholinesterase inhibition in ToxCast (Sipes et al., 2013), previous work has suggested that these assays are unable to fully capture acetylcholinesterase inhibition potency (Aylward and Hays, 2011). For these reasons, it was recommended that carbamates and organophosphates be excluded from this type of analysis. Further research identifying other biological perturbations and associated assays not covered by ToxCast will aid to reduce the uncertainty with toxicological space and minimize the application of exclusion criteria.

Another limitation of this approach that is more critical is the inability of the qHTS assays to accurately assess genotoxicity. Within ToxCast, there are only a few select assays that measure some component of DNA damage or repair to provide a prediction of genotoxic potential. Specifically, five assays have been identified that can detect stalled replication forks and/or DNA double-strand breaks. However, these assays have low sensitivity for predicting genotoxic potency, with only 40% of known, direct-acting genotoxic chemicals displaying activity in one or more of the assays related to genotoxicity (Hsieh et al., 2019). This analysis was restricted to chemicals known to be positive without metabolic activation. Considering that many mutagens are pro-mutagenic, in that metabolic activation is a requirement for genotoxicity, the sensitivity could potentially be lower, as the assays preclude the use of rat liver S9 required for metabolic competency. Thus, genotoxicity assessment is currently beyond the scope of this approach.

A parallel approach or testing strategy that uses *in silico* models (e.g., Pradeep et al., 2021)) and *in vitro* NAM data for genotoxicity assessment is currently under development to support high-throughput screening efforts. Several new assays have been developed that greatly enhance the throughput, sensitivity, and mechanistic information in detecting genotoxic chemicals. Quantitative dose-response modeling can be applied to the *in vitro* data, and the genotoxic concentrations can be coupled with IVIVE to derive a POD_Genotoxicity_ in the same way that the POD_Bioactivity_ was derived here. The assays that hold promise include, but are not limited to, those that use flow cytometry to detect DNA damage directly (MicroFlow®) (Avlasevich et al., 2006; Bryce et al., 2010) or detect DNA damage response elements (MultiFlow®) (Bryce et al., 2018), use reporter cell lines to detect DNA damage response elements (ToxTracker®) (Hendriks et al., 2012), use transgenic cell lines to detect point mutations or insertions/deletions (indels) in mutation reporter transgenes (FE1 MutaMouse) (Maertens et al., 2017; White et al., 2003), or use gel electrophoresis and cell imaging to detect DNA strand breaks in single cell microwells (CometChip®) (Chao and Engelward, 2020; Weingeist et al., 2013). Apart from these assays, there are also lower throughput genomic-based NAMs that can comprehensively interrogate the mutagenic mechanisms of a chemical. Specifically, error-corrected next-generation sequencing technologies have been shown to detect somatic cell mutations with extreme accuracy (Salk et al., 2018; Salk and Kennedy, 2020; Schmitt et al., 2012). The analysis of transcriptomic biomarkers has also been shown to be a powerful tool for classifying genotoxic and DNA damage-inducing chemicals (Li et al., 2015, 2017). A combination of these assays in the assessment of chemical hazard could greatly benefit the application of NAM data in chemical screening and prioritization.

Incorporation of additional sources of genomic-based bioactivity data, including transcriptomics data targeting either the whole transcriptome or surrogate biomarker panels, could greatly enhance the biological space and complexity of the bioactivity estimates (Harrill et al., 2019, 2021). Specifically, a high-throughput transcriptomics (HTTr) approach based on RNA-seq of cell lysates can enable cost-efficient screening of thousands of chemicals (Thomas et al., 2019), rivaling the qHTS assays used in this approach. Similar to the POD_Bioactivity_, a nondescript aggregate transcriptomic POD could be derived using benchmark concentrations based on active genes or pathways following chemical exposures (Farmahin et al., 2017; Thomas et al., 2013b). Alternatively, differentially expressed genes associated with chemical exposure can be linked to key events in biological pathways within the AOP framework (Ankley et al., 2010; Villeneuve et al., 2014), allowing for the derivation of a POD based on a specific adverse outcome. The HTTr approach has the potential to study all known biological pathways indicative of chemical toxicity and offers an opportunity to identify and explore novel AOPs.

In order to use *in vitro* bioassay results in supporting hazard characterization or risk assessment decision-making, *in vivo* equivalent dose context is required. To achieve this, IVIVE of the bioactivity concentration, relating to the concentration at which a chemical may induce a hazard, was performed using a generic HTTK model. The generic model is more advantageous than chemical-specific models, as its application can be extended to a diverse chemical space, such as that of the DSL, with more confidence (Wambaugh et al., 2015, 2018). To run the model, certain *in vitro* parameters are required, and these data are missing for many DSL chemicals; thus, *in silico* predictions were applied. It is acknowledged that these *in silico* predictions increase the uncertainty of the approach; it is also recognized that HTTK may not be suitable for certain chemicals, such as those that bioaccumulate and fail to reach steady state (Wambaugh et al., 2015). For these reasons, filters were applied to eliminate chemicals from the analysis that may not give suitable parameters or may not be appropriate for the generic model. This constrained the number of chemicals to which HTTK modeling could be applied but increased the confidence in model implementation. Comparing C_ss_ values derived using *in vitro* parameters with C_ss_ values derived using *in silico* parameters demonstrated that most predictions were in the same order of magnitude as the expected value. Discrepant results do not necessarily suggest that the *in silico* predictions were poor; considering that *in silico* models may be trained using *in vivo* data, the *in silico* parameters could actually be more consistent with what would be expected *in vivo*. Further work establishing chemical groupings and determining the HTTK model assumptions that are most appropriate for those groupings in a decision tree framework will greatly enhance the accuracy of the IVIVE approach. Overall, the use of *in silico* predictions with HTTK was essential for the derivation of POD_Bioactivity_ values for a broad range of DSL chemicals, greatly extending the utility of this approach.

For the vast majority of DSL chemicals, limited to no hazard data is available. Thus, prioritization efforts are most often focused on the chemical space for which the greatest amount of traditional data exists as opposed to expanding the screening to inform broader activities including further scoping, information gathering, and targeted data generation to proactively increase knowledge related to the potential for hazard and risk. The ToxCast bioassay database contains toxicological endpoints for thousands of chemicals, many of which have structural similarity to chemicals on the DSL (Fig. S68). Although toxicity data is missing for many chemicals, the overlap in chemical space between ToxCast and the DSL provided an opportunity to source bioactivity data from chemicals in ToxCast and apply them to DSL chemicals that shared similar structural features. In this work, we explored using a GenRA approach to derive surrogate PODs for DSL chemicals lacking bioactivity data. The results showed that the POD_Read-Across_ was in the same order of magnitude as the true POD_Bioactivity_ for the majority of chemicals (79%). Thus, application of the POD_Read-Across_ can be viewed as a useful tool and an early step toward the identification of possible high-hazard chemicals that would otherwise be ignored in prioritization efforts. For the chemicals with the greatest difference between POD_Read-Across_ and POD_Bioactivity_, the POD_Read-Across_ tended to be higher than the POD_Bioactivity_ rather than lower (Fig. 5). Thus, chemicals with POD_Read-Across_ values are less likely to be identified as priorities than chemicals with a POD_Bioactivity_. However, this should not be viewed as a loss of information, as these chemicals would routinely be excluded from priority setting because of their lack of data. Although read-across is a well-established method, there is inherent uncertainty in the approach. The level of acceptability of uncertainty, irrespective of whether it is a traditional or a GenRA-based read-across, is generally dependent on the regulatory decision-making context. With any read-across method, the key sources of uncertainty are the choice of analogs and the nature of the data. In this study, analog selection was based on structural similarity analysis using mechanistically-based ToxPrint fingerprints. To address the uncertainty around the read-across approach, caution should be applied when interpreting POD_Read-Across_ values. Specifically, expert multi-disciplinary judgment should be used to confirm the appropriateness of analogs used to derive POD_Read-Across_ values for chemicals where the BER is low.

Additional approaches, such as the development and application of machine learning algorithms, should be explored to improve the prediction of bioactivity for chemicals lacking qHTS data and broaden the application of the BER approach. These algorithms could also be used to generate predictions for chemicals in ToxCast that have only been tested across a limited number of assays. For example, consensus models have been trained using ToxCast data to make categorical or continuous predictions on a chemical’s potential to interact with endocrine or androgen receptors (Mansouri et al., 2020, 2016). In order to train robust models for making bioactivity predictions, a sufficient level of balanced data with a sufficient number of positive and negative chemicals for a given endpoint is required. It is important to note that the data will not be sufficient for most endpoints. However, the most active assays in ToxCast should have adequate data that could be leveraged to train additional models. Establishing models that make confident predictions with a high balanced accuracy and have a well-defined domain of applicability will enhance the computational workflow for deriving BERs of data-poor chemicals.

When assessing risk, the characterization of chemical exposure levels in the population is equally as important as the hazard assessment. In this work, high-throughput exposure estimates were used as the denominator in the BER derivation, as these values were available for many chemicals on the DSL. One area of refinement to improve this workflow would be to use exposure levels from analyses conducted in the jurisdiction of the chemical inventory. For example, chemical exposure levels in the Canadian population, from environmental media, biomonitoring, or consumer products, would be more relevant to the prioritization of the DSL. Recent advancements in non-targeted biomonitoring have allowed the identification of chemicals of emerging concern present in the “exposome” (Dennis et al., 2017; Pourchet et al., 2020). Non-targeted biomonitoring and qHTS data can be viewed as complementary, and there is an opportunity to leverage both sources of information to identify chemicals of potentially higher risk detected in human populations (Rager et al., 2016). One vision for future application could be that the POD_Bioactivity_ or POD_Read-Across_ values are used to identify the chemicals with higher hazard potential present in the exposome, supporting more targeted biomonitoring efforts to be used in the context of risk assessment.

Another consideration for risk-based prioritization is that many chemicals have no known exposure levels; however, many of these chemicals could have functional properties that make them suitable substitutes for chemicals undergoing risk management. For example, several analogs to the known endocrine disruptor bisphenol A exist on the DSL, and such analogues have been detected in Canadian house dust (Fan et al., 2021), highlighting the rising concern about these replacements, and similarly for others across the broad chemical space, in commerce. Moreover, many chemicals without known exposure may have broad use applications that are known, and this information could enable exposure levels to be estimated. Thus, a lack of exposure data should not preclude chemicals from rapid screening efforts, and hazard and use potential should be considered in the problem formulation. One approach that shows promise for this purpose is the use of quantitative structure-use relationship (QSUR) models to identify potential chemical functional substitutes (Phillips et al., 2017). Together with qHTS data, the QSUR models could be used to flag chemicals in commerce that have higher risk potential so that they can be surveyed or monitored more strategically. Concerted and coordinated efforts to identify use scenarios and estimate exposure levels for these chemicals would enhance the protection of public health and prevent unnecessary animal use.

Here we have applied the BER approach (Paul Friedman et al., 2019) to the Canadian DSL to demonstrate the applicability of *in vitro* bioactivity data and *in silico* models for quantitative risk-based prioritization and assessment. The 5,801 PODs and 3,679 BERs derived using the computational workflow can be used as part of a weight-of-evidence approach, with other approaches such as the TTC and other quantitative structure-activity relationship models, such as the Conditional Toxicity Value predictor (Wignall et al., 2018), in accelerating the identification of emerging priorities for the protection of human health. It is envisioned that as NAMs advance and more confidence is established in these approaches the pace and transparency of chemical evaluation will be greatly improved, and more concentrated efforts can be placed on tiered testing and assessment of chemicals that are of greater potential concern.

## Supplementary Material

Supplement1

Supplement2

## Footnotes

1
https://pollution-waste.canada.ca/substances-search/Substance/SearchByListOrGroup?ListGroupCode=DSL&viewOnline=View+online

2
https://www.canada.ca/en/environment-climate-change/services/evaluating-existing-substances/identification-risk-assessment-priorities-irap-2017–18.html

3
https://comptox.epa.gov/dashboard/

4
https://www.r-project.org/

5
https://github.com/MarcBeal/HC-ESRAB/tree/master/DSL_Bioactivity_PODs

6 doi:10.14573/altex.2106171s1

7
https://rdrr.io/bioc/ChemmineOB/

8 doi:10.14573/altex.2106171s2

## References

[R1] Ankley GT, Bennett RS, Erickson RJ (2010). Adverse outcome pathways: A conceptual framework to support ecotoxicology research and risk assessment. Environ Toxicol Chem 29, 730–741. doi:10.1002/etc.34 20821501

[R2] Avlasevich SL, Bryce SM, Cairns SE (2006). In vitro micronucleus scoring by flow cytometry: Differential staining of micronuclei versus apoptotic and necrotic chromatin enhances assay reliability. Environ Mol Mutagen 47, 56–66. doi:10.1002/em.20170 16180205

[R3] Aylward LL and Hays SM (2011). Consideration of dosimetry in evaluation of ToxCast™ data. J Appl Toxicol 31, 741–751. doi:10.1002/jat.1626 21381051

[R4] Barter ZE, Bayliss MK, Beaune PH (2007). Scaling factors for the extrapolation of in vivo metabolic drug clearance from in vitro data: Reaching a consensus on values of human micro-somal protein and hepatocellularity per gram of liver. Curr Drug Metab 8, 33–45. doi:10.2174/138920007779315053 17266522

[R5] Blackwell BR, Ankley GT, Corsi SR (2017). An “EAR” on environmental surveillance and monitoring: A case study on the use of exposure-activity ratios (EARs) to prioritize sites, chemicals, and bioactivities of concern in great lakes waters. Environ Sci Technol 51, 8713–8724. doi:10.1021/acs.est.7b01613 28671818 PMC6132252

[R6] Bryce SM, Avlasevich SL, Bemis JC (2010). Miniaturized flow cytometric in vitro micronucleus assay represents an efficient tool for comprehensively characterizing genotoxicity dose-response relationships. Mutat Res 703, 191–199. doi:10.1016/j.mrgentox.2010.08.020 20826227 PMC2991389

[R7] Bryce SM, Bernacki DT, Smith-Roe SL (2018). Investigating the generalizability of the MultiFlow® DNA damage assay and several companion machine learning models with a set of 103 diverse test chemicals. Toxicol Sci 162, 146–166. doi:10.1093/toxsci/kfx235 29106658 PMC6059150

[R8] Cao Y, Charisi A, Cheng L. (2008). ChemmineR: A compound mining framework for R. Bioinformatics 24, 1733–1734. doi:10.1093/bioinformatics/btn307 18596077 PMC2638865

[R9] Chao C. and Engelward BP (2020). Applications of CometChip for environmental health studies. Chem Res Toxicol 33, 1528–1538. doi:10.1021/acs.chemrestox.9b00393 32519858 PMC7568917

[R10] Cohen Hubal EA, Richard A, Aylward L. (2010). Advancing exposure characterization for chemical evaluation and risk assessment. J Toxicol Environ Health B 13, 299–313. doi:10.1080/10937404.2010.483947 20574904

[R11] Corsi SR, De Cicco LA, Villeneuve DL (2019). Prioritizing chemicals of ecological concern in great lakes tributaries using high-throughput screening data and adverse outcome pathways. Sci Total Environ 686, 995–1009. doi:10.1016/j.scitotenv.2019.05.457 31412529

[R12] Dennis KK, Marder E, Balshaw DM (2017). Biomonitoring in the era of the exposome. Environ Health Perspect 125, 502–510. doi:10.1289/EHP474 27385067 PMC5381997

[R13] EFSA and WHO – European Food Safety Authority and World Health Organization (2016). Review of the threshold of toxicological concern (TTC) approach and development of new TTC decision tree. EFSA Supporting Publications 13, 1006E. doi:10.2903/sp.efsa.2016.EN-1006

[R14] Fan X, Katuri GP, Caza AA (2021). Simultaneous measurement of 16 bisphenol A analogues in house dust and evaluation of two sampling techniques. Emerging Contaminants 7, 1–9. doi:10.1016/j.emcon.2020.12.001

[R15] Farmahin R, Williams A, Kuo B. (2017). Recommended approaches in the application of toxicogenomics to derive points of departure for chemical risk assessment. Arch Toxicol 91, 2045–2065. doi:10.1007/s00204-016-1886-5 27928627 PMC5399047

[R16] Filer DL, Kothiya P, Setzer RW (2016). tcpl: The ToxCast pipeline for high-throughput screening data. Bioinformatics 33, 618–620. doi:10.1093/bioinformatics/btw680 27797781

[R17] Gannon AM, Moreau M, Farmahin R. (2019). Hexabromocyclododecane (HBCD): A case study applying tiered testing for human health risk assessment. Food Chem Toxicol 131, 110581. doi:10.1016/j.fct.2019.110581 31202941

[R18] Harrill J, Shah I, Setzer RW (2019). Considerations for strategic use of high-throughput transcriptomics chemical screening data in regulatory decisions. Curr Opin Toxicol 15, 64–75. doi:10.1016/j.cotox.2019.05.004 31501805 PMC6733036

[R19] Harrill JA, Everett LJ, Haggard DE (2021). High-throughput transcriptomics platform for screening environmental chemicals. Toxicol Sci 181, 68–89. doi:10.1093/toxsci/kfab009 33538836 PMC10194851

[R20] Canada Health (2016). Science Approach Document: Threshold of Toxicological Concern (TTC)-Based Approach for Certain Substances (existing substance risk assessment bureau, ed.). Government of Canada, Ottawa, Ontario, Canada. https://bit.ly/3I8p2Ho

[R21] Health Canada (2021). Science approach document – Bio-activity exposure ratio: Application in priority setting and risk assessment. Canada Gazette 155. https://www.canada.ca/content/dam/eccc/documents/pdf/pded/bioactivity-exposure-ratio/Science-approach-document-bioactivity-exposure-ratio.pdf

[R22] Helman G, Patlewicz G. and Shah I. (2019). Quantitative prediction of repeat dose toxicity values using GenRA. Regul Toxicol Pharmacol 109, 104480. doi:10.1016/j.yrtph.2019.104480 31550520 PMC8565596

[R23] Hendriks G, Atallah M, Morolli B. (2012). The ToxTracker assay: Novel GFP reporter systems that provide mechanistic insight into the genotoxic properties of chemicals. Toxicol Sci 125, 285–298. doi:10.1093/toxsci/kfr281 22003191

[R24] Hsieh J, Smith-Roe SL, Huang R. (2019). Identifying compounds with genotoxicity potential using Tox21 high-throughput screening assays. Chem Res Toxicol 32, 1384–1401. doi:10.1021/acs.chemrestox.9b00053 31243984 PMC6740247

[R25] Johnson CL, Dohrmann SM, Burt VL (2014). National health and nutrition examination survey: Sample design, 2011–2014. Vital Health Stat 2, 1–33. 25569458

[R26] Judson RS, Kavlock RJ, Setzer RW (2011). Estimating toxicity-related biological pathway altering doses for high-throughput chemical risk assessment. Chem Res Toxicol 24, 451–462. doi:10.1021/tx100428e 21384849

[R27] Kavlock RJ, Bahadori T, Barton-Maclaren TS (2018). Accelerating the pace of chemical risk assessment. Chem Res Toxicol 31, 287–290. doi:10.1021/acs.chemrestox.7b00339 29600706 PMC6666390

[R28] Kroes R, Renwick A, Cheeseman M. (2004). Structure-based thresholds of toxicological concern (TTC): Guidance for application to substances present at low levels in the diet. Food Chem Toxicol 42, 65–83. doi:10.1016/j.fct.2003.08.006 14630131

[R29] Li H, Hyduke DR, Chen R. (2015). Development of a toxicogenomics signature for genotoxicity using a dose-optimization and informatics strategy in human cells. Environ Mol Mutagen 56, 505–519. doi:10.1002/em.21941 25733355 PMC4506269

[R30] Li HH, Chen R, Hyduke DR (2017). Development and validation of a high-throughput transcriptomic biomarker to address 21^st^ century genetic toxicology needs. Proc Natl Acad Sci USA 114, E10881–E10889. doi:10.1073/pnas.1714109114 29203651 PMC5754797

[R31] Lipinski CA, Lombardo F, Dominy BW (1997). Experimental and computational approaches to estimate solubility and permeability in drug discovery and development settings. Adv Drug Deliv Rev 23, 3–25. doi:10.1016/s0169-409x(00)00129-0 11259830

[R32] Maertens RM, Long AS and White PA (2017). Performance of the in vitro transgene mutation assay in MutaMouse FE1 cells: Evaluation of nine misleading (“false”) positive chemicals. Environ Mol Mutagen 58, 582–591. doi:10.1002/em.22125 28843037

[R33] Mansouri K, Abdelaziz A, Rybacka A. (2016). CERAPP: Collaborative estrogen receptor activity prediction project. Environ Health Perspect 124, 1023–1033. doi:10.1289/ehp.1510267 26908244 PMC4937869

[R34] Mansouri K, Kleinstreuer N, Abdelaziz AM (2020). CoMPARA: Collaborative modeling project for androgen receptor activity. Environ Health Perspect 128, 027002. doi:10.1289/EHP5580 PMC7064318 32074470

[R35] O’Boyle NM, Banck M, James CA (2011). Open Babel: An open chemical toolbox. J Cheminform 3, 33. doi: 10.1186/1758-2946-3-33 21982300 PMC3198950

[R36] OECD (2019). International Best Practices for Identification of Priorities within Chemicals Management Systems. OECD Series on Testing and Assessment, No. 314. OECD Publishing, Paris. 10.1787/0fafd6f5-en

[R37] Patlewicz G, Jeliazkova N, Safford R. (2008). An evaluation of the implementation of the Cramer classification scheme in the Toxtree software. SAR QSAR Environ Res 19, 495–524. doi:10.1080/10629360802083871 18853299

[R38] Patlewicz G, Kuseva C, Kesova A. (2014). Towards AOP application – Implementation of an integrated approach to testing and assessment (IATA) into a pipeline tool for skin sensitization. Regul Toxicol Pharmacol 69, 529–545. doi:10.1016/j.yrtph.2014.06.001 24928565

[R39] Patlewicz G, Wambaugh JF, Felter SP (2018). Utilizing threshold of toxicological concern (TTC) with high throughput exposure predictions (HTE) as a risk-based prioritization approach for thousands of chemicals. Comput Toxicol 7, 58–67. doi:10.1016/j.comtox.2018.07.002 31338483 PMC6650166

[R40] Paul Friedman K, Papineni S, Marty MS (2016). A predictive data-driven framework for endocrine prioritization: A triazole fungicide case study. Crit Rev Toxicol 46, 785–833. doi: 10.1080/10408444.2016.1193722 27347635 PMC5044773

[R41] Paul Friedman K, Gagne M, Loo L. (2019). Utility of in vitro bioactivity as a lower bound estimate of in vivo adverse effect levels and in risk-based prioritization. Toxicol Sci 173, 202–225. doi:10.1093/toxsci/kfz201 PMC7720780 31532525

[R42] Pearce RG, Setzer RW, Strope CL (2017). httk: R package for high-throughput toxicokinetics. J Stat Softw 79, 1–26. doi:10.18637/jss.v079.i04 30220889 PMC6134854

[R43] Phillips KA, Wambaugh JF, Grulke CM (2017). High-throughput screening of chemicals as functional substitutes using structure-based classification models. Green Chem 19, 1063–1074. doi:10.1039/C6GC02744J 30505234 PMC6260937

[R44] Pourchet M, Debrauwer L, Klanova J. (2020). Suspect and non-targeted screening of chemicals of emerging concern for human biomonitoring, environmental health studies and support to risk assessment: From promises to challenges and harmonisation issues. Environ Int 139, 105545. doi:10.1016/j.envint.2020.105545 32361063

[R45] Pradeep P, Patlewicz G, Pearce R. (2020). Using chemical structure information to develop predictive models for in vitro toxicokinetic parameters to inform high-throughput risk-assessment. Comput Toxicol 16, 100136. doi:10.1016/j.comtox.2020.100136 PMC8193769 34124416

[R46] Pradeep P, Judson R, DeMarini DM (2021). An evaluation of existing QSAR models and structural alerts and development of new ensemble models for genotoxicity using a newly compiled experimental dataset. Comput Toxicol 18, 100167. doi:10.1016/j.comtox.2021.100167 PMC8422876 34504984

[R47] Rager JE, Strynar MJ, Liang S. (2016). Linking high resolution mass spectrometry data with exposure and toxicity forecasts to advance high-throughput environmental monitoring. Environ Int 88, 269–280. doi:10.1016/j.envint.2015.12.008 26812473

[R48] Rajkumar A, Luu T, Beal MA (2021). Elucidation of the effects of bisphenol A and structural analogs on germ and steroidogenic cells using single cell high-content imaging. Toxicol Sci 180, 224–238. doi:10.1093/toxsci/kfab012 33501994 PMC8041462

[R49] Richard AM, Judson RS, Houck KA (2016). ToxCast chemical landscape: Paving the road to 21^st^ century toxicology. Chem Res Toxicol 29, 1225–1251. doi:10.1021/acs.chemrestox.6b00135 27367298

[R50] Ring CL, Pearce RG, Setzer RW (2017). Identifying populations sensitive to environmental chemicals by simulating toxicokinetic variability. Environ Int 106, 105–118. doi:10.1016/j.envint.2017.06.004 28628784 PMC6116525

[R51] Salk JJ, Schmitt MW and Loeb LA (2018). Enhancing the accuracy of next-generation sequencing for detecting rare and subclonal mutations. Nat Rev Genet 19, 269. doi:10.1038/nrg.2017.117 29576615 PMC6485430

[R52] Salk JJ and Kennedy SR (2020). Next-generation genotoxicology: Using modern sequencing technologies to assess somatic mutagenesis and cancer risk. Environ Mol Mutagen 61, 135–151. doi:10.1002/em.22342 31595553 PMC7003768

[R53] Schmitt MW, Kennedy SR, Salk JJ (2012). Detection of ultra-rare mutations by next-generation sequencing. Proc Natl Acad Sci U S A 109, 14508–14513. doi:10.1073/pnas.1208715109 22853953 PMC3437896

[R54] Shah I, Liu J, Judson RS (2016). Systematically evaluating read-across prediction and performance using a local validity approach characterized by chemical structure and bioactivity information. Regul Toxicol Pharmacol 79, 12–24. doi:10.1016/j.yrtph.2016.05.008 27174420

[R55] Sipes NS, Martin MT, Kothiya P. (2013). Profiling 976 ToxCast chemicals across 331 enzymatic and receptor signaling assays. Chem Res Toxicol 26, 878–895. doi:10.1021/tx400021f 23611293 PMC3685188

[R56] Sipes NS, Wambaugh JF, Pearce R. (2017). An intuitive approach for predicting potential human health risk with the Tox21 10k library. Environ Sci Technol 51, 10786–10796. doi:10.1021/acs.est.7b00650 28809115 PMC5657440

[R57] Steinbeck C, Han Y, Kuhn S. (2003). The chemistry development kit (CDK): An open-source java library for chemo-and bioinformatics. J Chem Inf Comput Sci 43, 493–500. doi:10.1021/ci025584y 12653513 PMC4901983

[R58] Thomas RS, Philbert MA, Auerbach SS (2013a). Incorporating new technologies into toxicity testing and risk assessment: Moving from 21^st^ century vision to a data-driven framework. Toxicol Sci 136, 4–18. doi:10.1093/toxsci/kft178 23958734 PMC3829570

[R59] Thomas RS, Wesselkamper SC, Wang NCY (2013b). Temporal concordance between apical and transcriptional points of departure for chemical risk assessment. Toxicol Sci 134, 180–194. doi:10.1093/toxsci/kft094 23596260

[R60] Thomas RS, Bahadori T, Buckley TJ (2019). The next generation blueprint of computational toxicology at the US environmental protection agency. Toxicol Sci 169, 317–332. doi:10.1093/toxsci/kfz058 30835285 PMC6542711

[R61] Tilley SK, Reif DM and Fry RC (2017). Incorporating ToxCast and Tox21 datasets to rank biological activity of chemicals at superfund sites in North Carolina. Environ Int 101, 19–26. doi:10.1016/j.envint.2016.10.006 28153528 PMC5351294

[R62] Tonnelier A, Coecke S. and Zaldívar J. (2012). Screening of chemicals for human bioaccumulative potential with a physiologically based toxicokinetic model. Arch Toxicol 86, 393–403. doi:10.1007/s00204-011-0768-0 22089525 PMC3282909

[R63] Turley AE, Isaacs KK, Wetmore BA (2019). Incorporating new approach methodologies in toxicity testing and exposure assessment for tiered risk assessment using the RISK21 approach: Case studies on food contact chemicals. Food Chem Toxicol 134, 110819. doi:10.1016/j.fct.2019.110819 31545997 PMC7429715

[R64] US EPA – US Environmental Protection Agency (2015). ToxCast & Tox21 MySQL database invitrodb (version 3).

[R65] Villeneuve DL, Crump D, Garcia-Reyero N. (2014). Adverse outcome pathway (AOP) development I: Strategies and principles. Toxicol Sci 142, 312–320. doi:10.1093/toxsci/kfu199 25466378 PMC4318923

[R66] Wambaugh JF, Setzer RW, Reif DM (2013). High-throughput models for exposure-based chemical prioritization in the ExpoCast project. Environ Sci Technol 47, 8479–8488. doi:10.1021/es400482g 23758710

[R67] Wambaugh JF, Wetmore BA, Pearce R. (2015). Toxicokinetic triage for environmental chemicals. Toxicol Sci 147, 55–67. doi:10.1093/toxsci/kfv118 26085347 PMC4560038

[R68] Wambaugh JF, Hughes MF, Ring CL (2018). Evaluating in vitro-in vivo extrapolation of toxicokinetics. Toxicol Sci 163, 152–169. doi:10.1093/toxsci/kfy020 29385628 PMC5920326

[R69] Weingeist DM, Ge J, Wood DK (2013). Single-cell microarray enables high-throughput evaluation of DNA double-strand breaks and DNA repair inhibitors. Cell Cycle 12, 907–915. doi:10.4161/cc.23880 23422001 PMC3637349

[R70] Wetmore BA, Wambaugh JF, Ferguson SS (2011). Integration of dosimetry, exposure, and high-throughput screening data in chemical toxicity assessment. Toxicol Sci 125, 157–174. doi:10.1093/toxsci/kfr254 21948869

[R71] Wetmore BA, Wambaugh JF, Ferguson SS (2013). Relative impact of incorporating pharmacokinetics on predicting in vivo hazard and mode of action from high-throughput in vitro toxicity assays. Toxicol Sci 132, 327–346. doi:10.1093/toxsci/kft012 23358191

[R72] Wetmore BA (2015). Quantitative in vitro-to-in vivo extrapolation in a high-throughput environment. Toxicology 332, 94–101. doi:10.1016/j.tox.2014.05.012 24907440

[R73] White PA, Douglas GR, Gingerich J. (2003). Development and characterization of a stable epithelial cell line from muta™ mouse lung. Environ Mol Mutagen 42, 166–184. doi:10.1002/em.10185 14556224

[R74] Wignall JA, Muratov E, Sedykh A. (2018). Conditional toxicity value (CTV) predictor: An in silico approach for generating quantitative risk estimates for chemicals. Environ Health Perspect 126, 057008. doi:10.1289/EHP2998 29847084 PMC6071978

[R75] Williams AJ, Grulke CM, Edwards J. (2017). The CompTox chemistry dashboard: A community data resource for environmental chemistry. J Cheminform 9, 61. doi:10.1186/s13321-017-0247-6 29185060 PMC5705535

[R76] Yang C, Tarkhov A, Marusczyk J. (2015). New publicly available chemical query language, CSRML, to support chemotype representations for application to data mining and modeling. J Chem Inf Model 55, 510–528. doi:10.1021/ci500667v 25647539

