# Supplementary material for: Implementing In Vitro Bioactivity Data to Modernize Priority Setting of Chemical Inventories
Source: ALTEX. Author manuscript; Available in PMC 2023 Jan 1. (PMC8973434; doi:10.14573/altex.2106171)
Supplement: Supplement1 [file NIHMS1778532-supplement-Supplement1.pdf]

Beal et al.:

Implementing *In Vitro* Bioactivity Data to Modernize Priority Setting of Chemical Inventories

Supplementary Data

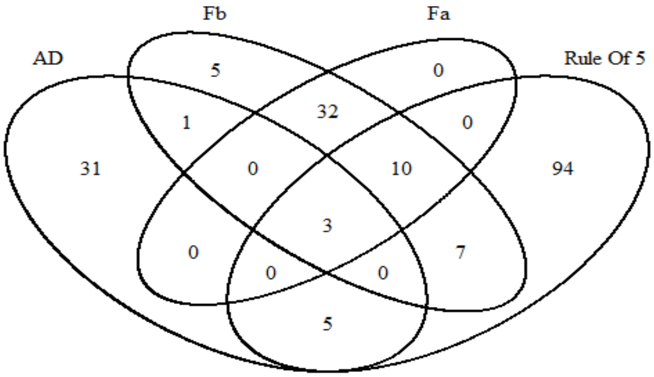

Fig. S1: Number of chemicals removed by each HTTK filter
The filters applied from left to right are applicability domain (AD), fraction bioavailable (Fb), fraction absorbed (Fa), and Lipinski's rule of five.

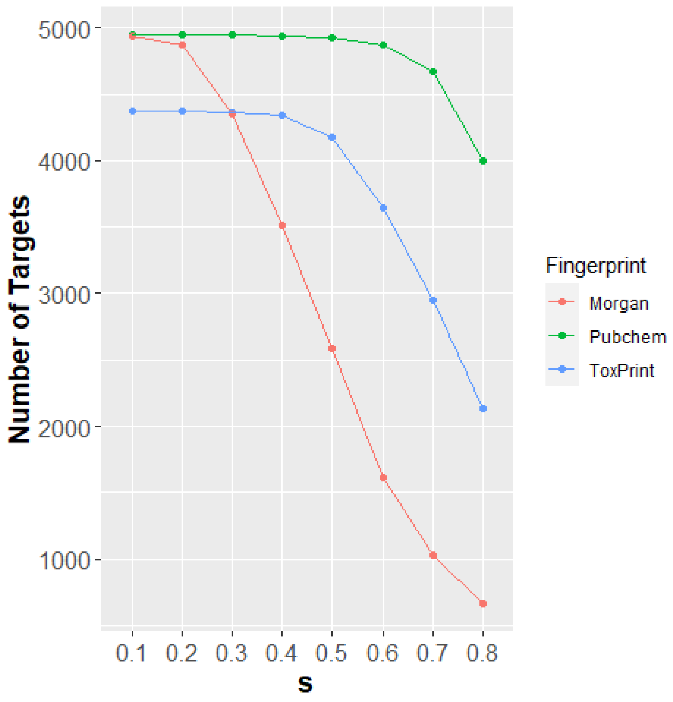

Fig. S2: Number of targets where read-across could be applied across different fingerprint types and s-values

doi:10.14573/altex.2106171s2

This is an Open Access article distributed under the terms of the Creative Commons Attribution 4.0 International license (http://creativecommons.org/licenses/by/4.0/), which permits unrestricted use, distribution and reproduction in any medium, provided the original work is appropriately cited.

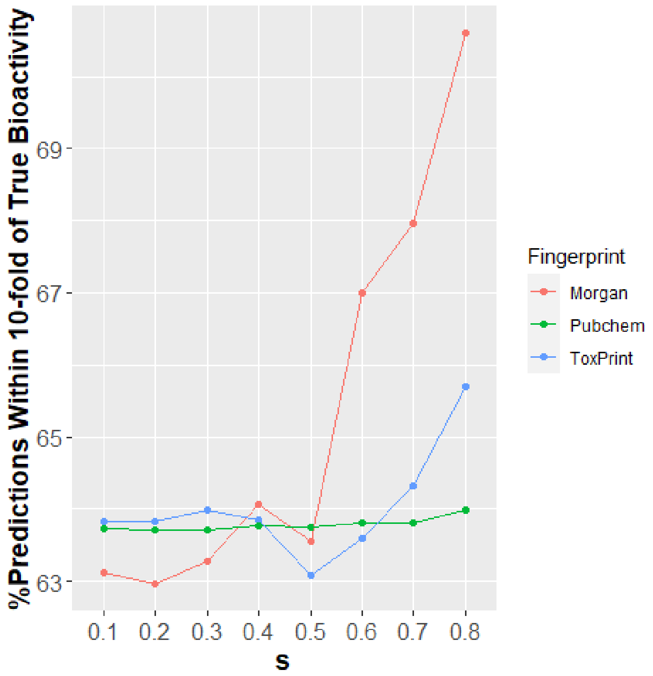

Fig. S3: Percent of targets with read-across bioactivity within 10-fold of true bioactivity across different fingerprint types and s-values

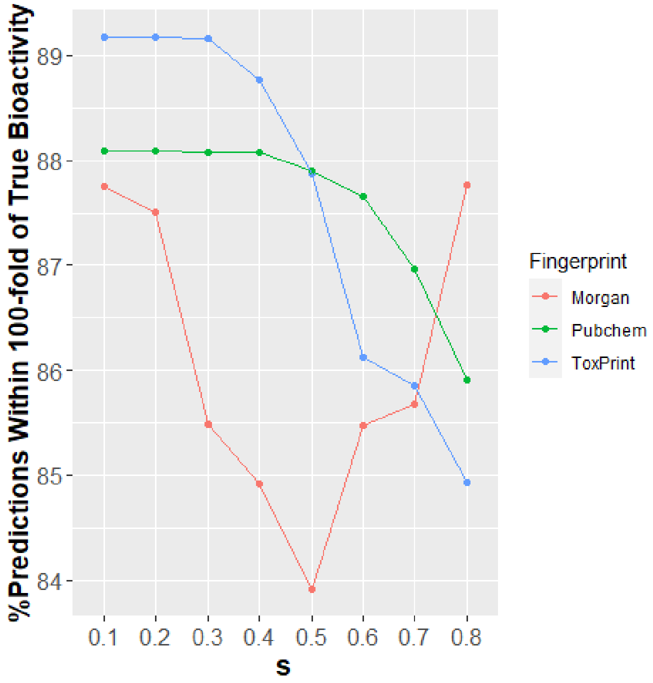

Fig. S4: Percent of targets with read-across bioactivity within 100-fold of true bioactivity across different fingerprint types and s-values

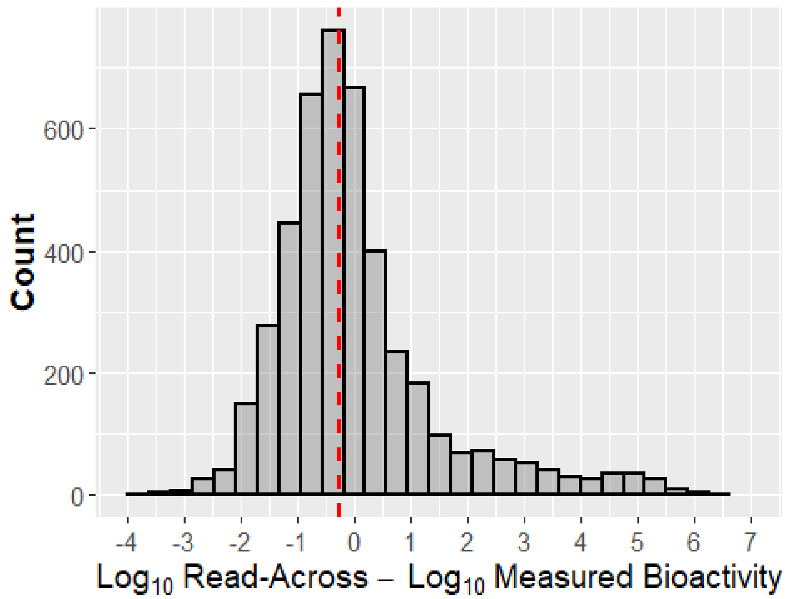

Fig. S5: Comparison of read-across bioactivity concentration with true bioactivity concentration derived from ToxCast data
For each chemical, the ToxCast bioactivity concentration was extracted (empirical concentration), and, where possible, bioactivity concentrations were derived for these chemicals using GenRA. The majority of chemicals (89.17%) have read-across values within 100-fold of the derived bioactivity concentration (adjusted r^2 = 0.0641).

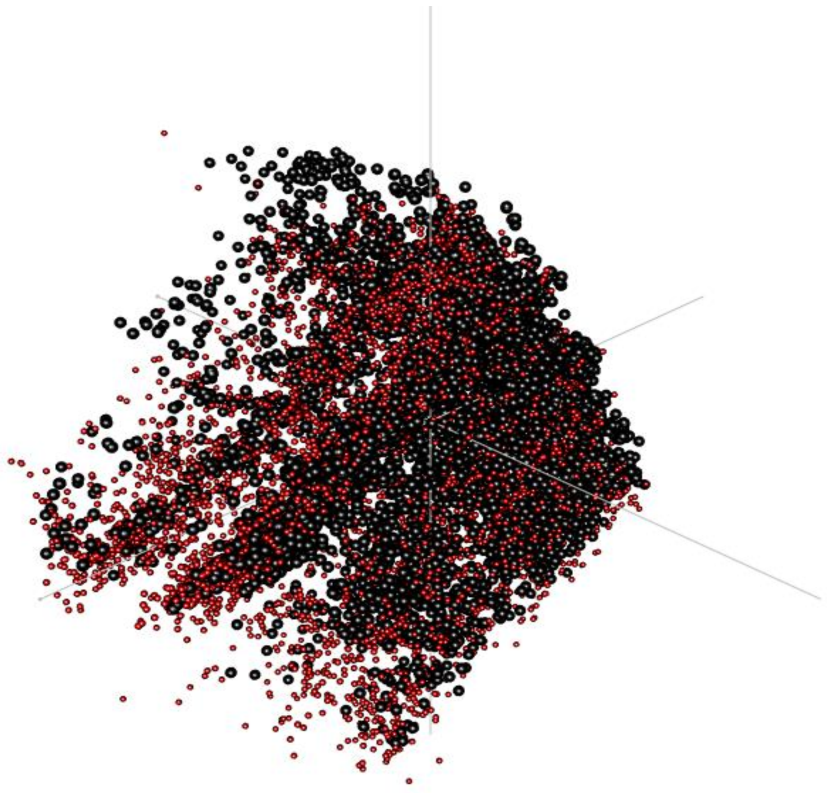

Fig. S6: Visualization of ToxCast and DSL chemical space
Classical multi-dimensional scaling (Gower, 1966) was used to visualize in three-dimensional space the comparison of ToxCast (black spheres) and DSL (red spheres) chemical space. DSL chemicals on the periphery have the lowest structural similarity to ToxCast chemicals and are less likely to have analogues for read-across (i.e., Tanimoto coefficient ≥ 0.3).

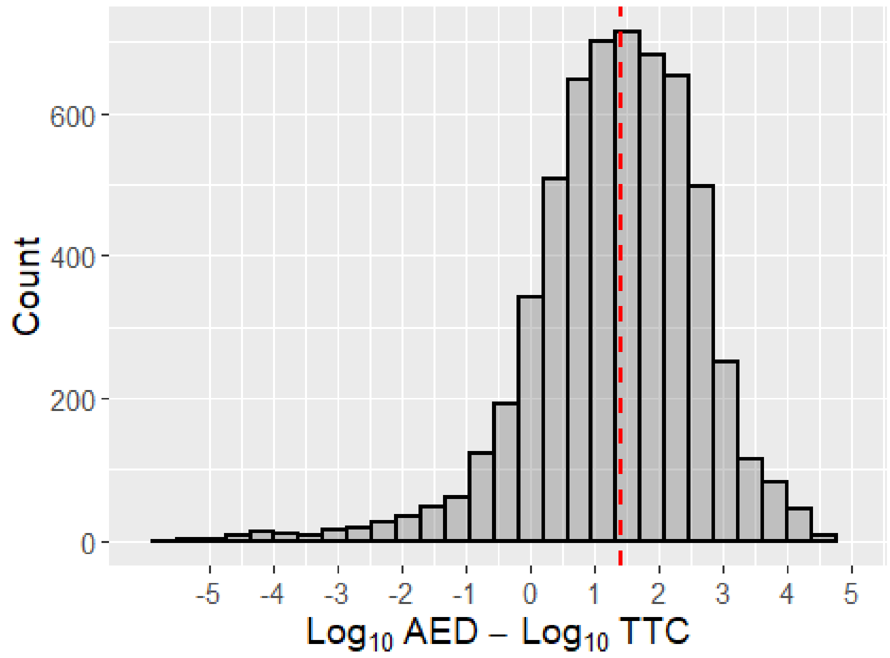

Fig. S7: Comparison of $POD_{Bioactivity}$ or $POD_{Read\text{-}Across}$ with TTC value
For the majority of chemicals (87.67%), the TTC is lower than the POD (adjusted r^2 = 0.0002).

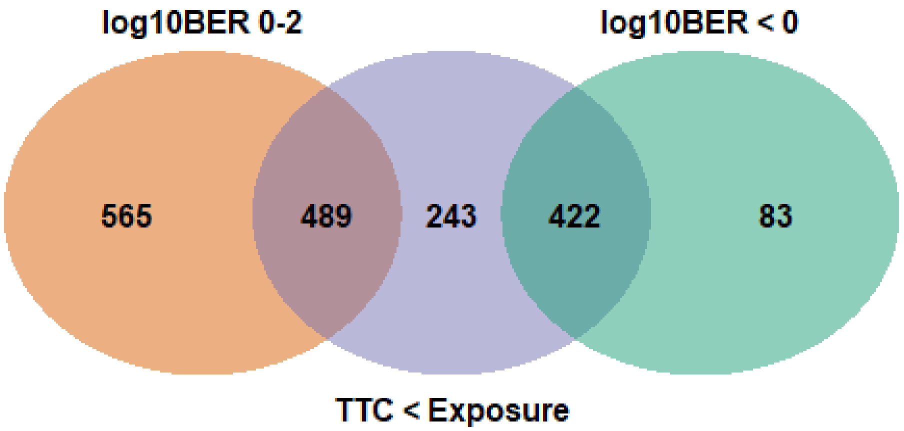

Fig. S8: Venn diagram comparing chemicals where exposure estimates were greater than TTC with chemicals with low BERs

Tab. S1: HTTK filters and their impact on number of chemicals removed and accuracy of prediction

	Chemicals removed	% C_{ss} within 10-fold	% C_{ss} within 10-fold
Unfiltered	0	75.94	94.31
Applicability domain filter	40	75.76	94.16
Lipinski's rule of five filter	119	78.45	95.57
Fraction absorbed filter	45	77.43	95.37
Fraction bioavailable filter	58	77.89	95.88
All filters	188	79.68	96.64

Tab. S2: List of chemicals with discrepancies between *in vitro*-derived C_{ss} **and *in silico* derived-** C_{ss} **that remained after filtering**

CASRN	Chemical	C_{ss}	*in silico*-derived C_{ss}	Log_{10} difference
486-56-6	Cotinine	43659.05	21.94	-3.30
95-69-2	4-chloro-2-methylaniline	7487.96	4.05	-3.27
120-32-1	Clorophene	9420.65	7.47	-3.10
100-01-6	4-nitroaniline	4280.37	7.46	-2.76
138472-01-2	Fr900409	2799.81	5.17	-2.73
50-52-2	Thioridazine	43.17	0.10	-2.66
62-73-7	Dichlorvos	1601.31	3.59	-2.65
654055-01-3	Morin hydrate	23704.59	79.13	-2.48
122-66-7	1,2-diphenylhydrazine	2579.79	8.87	-2.46
33089-61-1	Amitraz	193.33	0.79	-2.39
64706-54-3	Bepridil	78.88	0.38	-2.32
17804-35-2	Benomyl	1491.46	12.52	-2.08
75530-68-6	Nilvadipine	665.42	5.79	-2.06
58-38-8	Prochlorperazine	178.97	1.60	-2.05
69-09-0	Chlorpromazine hydrochloride	127.45	15300.69	2.08
17696-62-7	Phenylparaben	0.32	38.97	2.08
188489-07-8	Flufenpyr-ethyl	0.25	30.76	2.09
54593-83-8	Chlorethoxyfos	1.35	194.25	2.16
35575-96-3	Azamethiphos	0.02	3.52	2.23
321-64-2	Tacrine	0.02	3.67	2.27
156-10-5	4-nitrosodiphenylamine	0.13	28.89	2.34
136-45-8	Dipropyl pyridine-2,5-dicarboxylate	0.08	19.50	2.38
136-60-7	Butyl benzoate	0.16	40.38	2.40
55406-53-6	3-iodo-2-propynyl-n-butylcarbamate	0.04	17.76	2.68
105-87-3	Geranyl acetate	0.40	225.30	2.75

Tab. S3: Summary of POD comparisons for each response type

Response type	POD comparisons	Protective	Non-protective	Percent protective
Lowest traditional POD	2248	2077	171	92.39%
NOAEL	2230	2068	162	92.74%
BMDL	96	76	20	79.17%
LOAEL	1218	1149	69	94.33%
Lowest traditional POD filtered	1042	992	50	95.20%
NOAEL filtered	1037	990	47	95.47%
BMDL filtered	42	37	5	88.10%
LOAEL filtered	610	591	19	96.89%

Reference

Gower, J. C. (1966). Some distance properties of latent root and vector methods used in multivariate analysis. *Biometrika 53*, 325-338. doi:10.1093/biomet/53.3-4.325
